# Tailoring spin mixtures by ion-enhanced Maxwell magnetic coupling in color-tunable organic electroluminescent devices

**DOI:** 10.1038/s41377-018-0046-5

**Published:** 2018-08-01

**Authors:** Junwei Xu, Yue Cui, Gregory M. Smith, Peiyun Li, Chaochao Dun, Linqi Shao, Yang Guo, Hongzhi Wang, Yonghua Chen, David L. Carroll

**Affiliations:** 1 Center for Nanotechnology and Molecular Materials, Wake Forest University, Winston-Salem, NC 27109 USA; 2 Department of Physics, Wake Forest University, Winston-Salem, NC 27106 USA; 3 Key Laboratory of Luminescence and Optical Information (Ministry of Education), Institute of Optoelectronics Technology, Beijing Jiaotong University, 100044 Beijing, P.R. China; 4 Department of Electro-Optics, School of Engineering, University of Dayton, Dayton, OH 45469 USA; 5 Department of Mathematics, Wake Forest University, Winston-Salem, NC 27106 USA; 6 State Key Laboratory for Modification of Chemical Fibers and Polymer Materials, 201620 Shanghai, P.R. China; 7 College of Materials Science and Engineering, Donghua University, 201620 Shanghai, P.R. China; 8 Institute of Advanced Materials, Nanjing Tech University, 211816 Nanjing, Jiangsu P.R. China; 9 Present Address: Department of Physics and Astronomy, College of Charleston, Charleston, SC 29424 USA

## Abstract

In this work, we show that the spin dynamics of excitons can be dramatically altered by Maxwell magnetic field coupling, together with an ion-enhanced, low-internal-splitting-energy organic semiconducting emitter. By employing a unique, alternating current (AC)-driven organic electroluminescent (OEL) device architecture that optimizes this magnetic field coupling, almost complete control over the singlet-to-triplet ratio (from fluorescent to phosphorescent emission in a single device) is realized. We attribute this spin population control to magnetically sensitive polaron–spin pair intersystem crossings (ISCs) that can be directly manipulated through external driving conditions. As an illustration of the utility of this approach to spin-tailoring, we demonstrate a simple hybrid (double-layer) fluorescence–phosphorescence (F–P) device using a polyfluorene-based emitter with a strong external Zeeman effect and ion-induced long carrier diffusion. Remarkable control over de-excitation pathways is achieved by controlling the device-driving frequency, resulting in complete emission blue–red color tunability. Picosecond photoluminescence (PL) spectroscopy directly confirms that this color control derives from the magnetic manipulation of the singlet-to-triplet ratios. These results may pave the way to far more exotic organic devices with magnetic-field-coupled organic systems that are poised to usher in an era of dynamic spintronics at room temperature.

## Organic optoelectronics: spin-state control

The light emission of an organic electroluminescent device can be tuned between blue fluorescence and red phosphorescence by using magnetic field effects to control the exciton population of singlet and triplet spins within the device. That’s the finding of a Chinese-US collaboration that use an alternating current (AC) power source to generate the required magnetic fields for the spin-state control inside a hybrid device featuring the blue fluorescent emitter polyfluorene (PFN-Br) and the red phosphorescent emitter PVK:Ir(MDQ)_2_(acac). By sweeping the frequency of the drive voltage from 50 to 70,000 Hz the color of the emission was seen to shift from blue to cold-white to warm-white to red. In addition to applications as a tunable light source, the approach could also be useful for performing experiments in dynamic spintronics at room temperature.

## Introduction

In today’s current-driven organic electroluminescence (OEL) devices, of which organic lighting diodes are a subset, free charges are injected through metal contacts into an organic semiconductor, where they can recombine into electron–hole (e–h) pairs and decay to emit photons^1,2^ (or they can be transported through the layer without interacting). Quantum statistics dictates that the fraction of spin pairs that are formed in the spin-triplet excited state is generally fixed at 75%. The spin-singlet states make up the remaining 25% of the excited-state population^3,4^. This means that when re-combining injected spin–1/2 polaron–spin pairs for EL, three of every four possible spin combinations will be triplets (with nonradiative or infrared decay routes), and one of the four will be a singlet (fluorescent emission). This has prompted the use of triplet scavenging dyes that resonantly transfer the triplet energy to a metal complex (e.g., Pt, Os, Ir, Au, Pd, or Ru) with strong L-S coupling^5–7^, which allows phosphorescent emission and higher device efficiency.

The resonant transfer of energy is useful for lighting and display applications. However, owing to the lifetimes involved in triplet transfer or decay, the limits on the current flux and dye concentrations due to co-localization of triplet energy, and the subsequent quenching, among other considerations, resonant energy transfer does not address the issues that spin-triplets cause for high-performance light-emitting applications. These potential but unrealized applications include the electrically stimulated organic laser^8^, the photonic spin valve, organically based optical computing paradigms, and telecommunications. Without more complete spin-population control, it is unlikely that any of these technologies will be realized using the organic platform^9^.

Until recently, there did not seem to be any way to beat the statistics of this problem. However, in 2007^10^, a hint was provided experimentally in the negative magnetoresistance of an organic semiconductor device. The typical 75%:25% ratio of triplets to singlets is not maintained when external, static magnetic fields are present. This has been attributed to a spin remixing in the polaron–spin pairs that is typically found in organic systems^10–13^. Simply, the external magnetic field can perturb the coherent relationship between electron and hole spin precessions^14–16^. Therefore, an enhanced singlet-related emission can be observed in a florescent semiconducting polymer that has a low internal splitting energy or a strong Zeeman effect. However, phosphorescent organic semiconductors do not exhibit the spin remixing property in the external magnetic field since the heavy metal atom can significantly raise the internal splitting energy of the organic compound^13^. Moreover, in magnetically coupled OEL devices, the redistribution of singlet and triplet e–h pairs can give rise to a significant change in the electrical current in the semiconductor through dissociation^17–21^ and charge reaction^22–25^ (Figure S1). However, the key to utility is to extend this control over the spin populations.

By utilizing the magnetic field spin remixing phenomena, in our present work, a novel device architecture is optimized to self-couple the time-dependent Maxwell field into the ion-assisted emitter. With a suitable choice of emitters for this structure, a high color contrast in spin-state ratios for a single compact device structure can be achieved. Color-tunable pixels have a unique potential for use in ultra-high-resolution information displays^26,27^ since they allow for an optimal fill factor and impart further momentum toward realizing high-definition micro-displays. One of most common color-tuning strategies is to induce a voltage-dependent color shift via a spatial shift of the recombination zone or exciton redistribution within a multi-layered device^28,29^. However, this leads to inevitable brightness changes and quenching in the high fields, making the voltage-dependent color-tunable devices more difficult and expensive to commercialize. Another method seen in the literature is to stack two or three color-independent OEL segments into a tandem device^27,30^. However, stacking tandem EL structures requires complicated multiple thin film deposition and designs of electrical power for multiple electrodes.

Here a color-change hybrid alternating current (AC)-driven OEL device is driven with a high-frequency AC electric field. This yields a Maxwell AC magnetic field that is coupled to the organic emitter, namely, a p–n-emitting interface. The internal AC magnetic field allows the manipulation of the ratio between singlet and triplet e–h pairs in an n-type fluorescent material by suppressing intersystem crossing (ISC) to generate diffusive secondary carriers. Then these ion-enhanced secondary carriers produce triplet-spin excitons in a proximal phosphorescent organic matrix. In the magnetically coupled fluorescence–phosphorescence (F–P) hybrid OEL device, we successfully shifted the CIE coordinates of the radiative output from (0.23, 0.34) to (0.53, 0.40) by manipulating the driving frequency from 50 to 60,000 Hz with no significant brightness change.

## Results

**Fig. 1:**
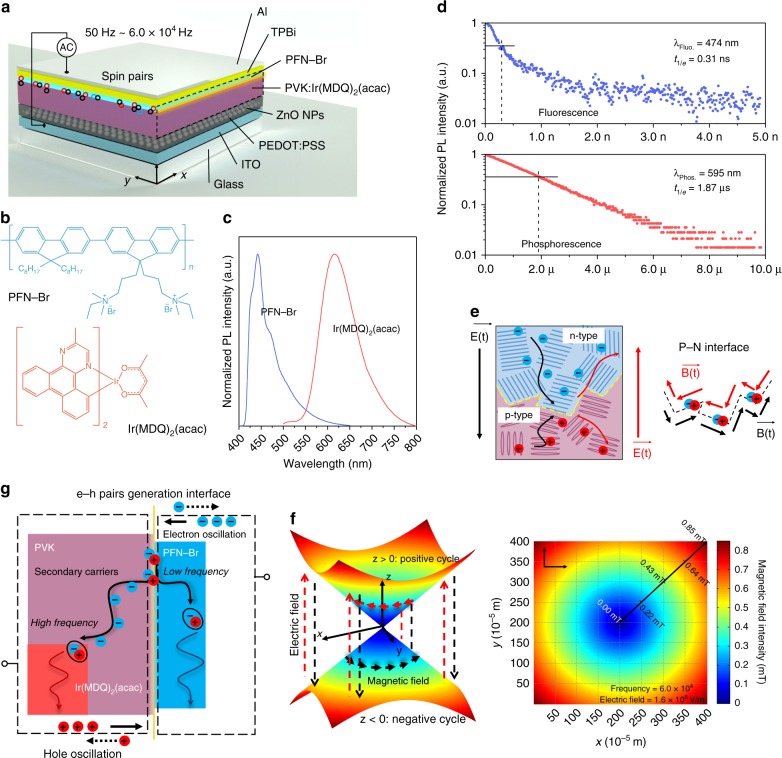
Structural and electromagnetic characterization of color-tunable AC-OEL devices. **a** Compositional schematic diagram of devices with a fluorescence–phosphorescence (F–P) emission interface driven by an AC power source (50–70,000 Hz). Singlet spins (blue circle pairs) and triplet spins (red circle pairs) are formed at the PVK/PFN-Br interface (or F–P interface). **b** The chemical structures of PFN-Br and Ir(MDQ)_2_(acac). **c** Fluorescence of PFN-Br at 474 nm and phosphorescence of Ir(MDQ)_2_(acac) at 595 nm. **d** Photoluminescence (PL) decay curves of PFN-Br fluorescent emission (top) and Ir(MDQ)_2_(acac) phosphorescent emission (bottom). **e** Schematic diagram of dynamic field analysis at the p–n magnetic interface. **f** Simulated results of magnetic and electric fields at the p–n junction at 60,000 Hz. The maximum self-generated magnetic field is 0.85 mT. In the positive and negative halves of the AC driving cycle, the directions of the vertex magnetic fields are clockwise (*z* > 0) and counterclockwise (*z* < 0), respectively. **g** Proposed working principle of the e–h pair generation interface. Radical triplet excitons are generated in Ir(MDQ)_2_(acac) by secondary carriers for red phosphorescent emission at high frequency. At low frequency, radical singlet excitons decay in PFN-Br for blue fluorescent emission

Our device architecture (Fig. 1a) borrows from the “gated” AC-OEL structure that were introduced recently in the literature^31^. In this case, however, the gated structure is coupled to a bilayer organic emitter consisting of [poly(N-vinylcarbazole) (PVK):bis(2-methyldibenzo[f,h]quinoxaline) (acetylacetonate)iridium(III) (Ir(MDQ)_2_(acac)) and poly[(9,9-bis(3’-((N,N-dimethyl)-N-ethylammonium)-propyl)-2,7-fluorene)-alt-2,7-(9,9-dioctylfluorene)] (PFN-Br)]. Surface morphology analyses of PVK:Ir(MDQ)_2_(acac) and PFN-Br emissive layers are performed via atomic force microscopy (AFM); the obtained images can be found in Figure S2. As in an earlier work^32^, the carrier gate layer was composed of [poly(3,4-ethylenedioxythiophene) polystyrene sulfonate (PEDOT:PSS) that was loaded with ZnO nanoparticles (NPs)] and the electron-transporting layer was [2,2′,2”-(1,3,5-benzinetriyl)-tris(1-phenyl-1-H-benzimidazole) (TPBi)]. The two conductive electrodes were indium tin oxide (ITO) and aluminum (Al). The chemical structures of the emitters, namely, PFN-Br and Ir(MDQ)_2_(acac), are listed in Fig. 1b. With 365 nm ultraviolet (UV) excitation, PFN-Br and Ir(MDQ)_2_(acac) show strong fluorescent and phosphorescent luminescence peaks at 474 and 585 nm, respectively (as shown in Fig. 1c). The lifetimes of short-lived blue fluorescence and long-lived red phosphorescence are given in Fig. 1d for 0.31 ns and 1.87 μs, respectively. With ZnO NPs (~35 nm in diameter) gating the interface of ITO/PEDOT:PSS, injected charges can be efficiently manipulated under the forward and reverse biases of AC cycles^32–34^. The AC-OEL devices are driven by a sinusoidal voltage with a wide frequency range from 50 Hz, which causes the gate to allow bi-polar injection and the device to act like a diode, to 60,000 Hz, at which most light is created by field-generated polarons/excitons with little diffusive transport in the active volume of the emitter. The operating mechanism of AC-OEL devices is described in detail in Supplemental Information.

PFN-Br is a high-performance ionized electron-transporting polymer^35,36^ with an electron mobility of 1.41 × 10^−^^7^ cm^2^/V/s, and PVK is a typical p-type semiconductor with a hole mobility on the order of 1.0 × 10^−6^ cm^2^/V/s. Thus electrons and holes are transferred and interact at the PVK/PFN-Br interface under an external electric field. The time for accumulation at this interface depends on the driving frequency. However, as already noted at high frequencies, the gate of the system allows only for field-generated carrier injection into the emitting volume, while at lower frequencies the gate allows for direct injection from the contacts. Nevertheless, both conditions result in charge drift toward the interface (illustrated in Fig. 1e). The electrons and holes are transferred to the heterointerface in the positive cycle of the AC electric field and drift along the opposite direction under the reverse bias^37–39^. Therefore, the time-dependent electric field generates an interfacial magnetic field at the PVK:Ir(MDQ)_2_(acac)/PFN-Br heterointerface according to Maxwell’s equations. In an ideal case, the pixel dimension is 4 × 4 mm^2^, which is significantly larger than its thickness (~300 nm); hence, it is reasonable to ignore fringing effects (the infinite-area parallel-plate-capacitor assumption). Via engaging a high-frequency driving (60,000 Hz) under a strong AC electrical field (1.6 × 10^8^ V/m), the temporal and spatial characteristics of the internal magnetic field are shown in Fig. 1f. The upper and lower half-planes represent the opposite “clock directions” of the magnetic field in the positive and negative halves of an AC cycle. The amplitude of the magnetic field is estimated to be approximately 0.85 mT. More details can be found in Supplemental Information, along with simulation results with various frequencies in Figure S3. The dynamic revolution of the magnetic field is shown in Movie S1.

The heterointerface also plays the role of an e–h pair recombination zone for hot carrier injection, as shown in the energy-level diagram in Fig. 1g. These e–h pairs not only move in the applied electric field but also experience the induced magnetic field. The device shows a blue emission (in Figure S4) due to the PFN-Br fluorescence at near-DC driving (50 Hz) since the dissociation rate of e–h pairs is negligible in the absence of an induced magnetic field (<0.00005 mT). When this internal AC magnetic field is of the same order as the nuclear hyperfine field (~1 mT), ISC suppression should occur^11^ and this would naturally lead to singlet-spin e–h pair accumulation. Many secondary carriers are produced in PFN-Br through the magnetically mediated dissociation of the e–h pairs. The secondary charges diffuse to nearby Ir(MDQ)_2_(acac) sites, which yields decay of triplet-state excitons, as shown in Figure S4. There is no significant position shift of the recombination zone in the device, as shown in Figure S5. Thus, as illustrated in Fig. 1g, in the low-frequency driving regime (50–1000 Hz), hot carrier injection is the main mechanism for fluorescent excitons in PFN-Br. In the high-frequency regime (30,000–70,000 Hz), the high-intensity AC magnetic field at the F–P interface populates singlet-excited e–h pairs mostly via ISC suppression, which leads to secondary carriers. The secondary carriers exist in the form of bonded electrons in the PFN-Br polymer matrix, more specifically, with Br atoms, which are strong electron acceptors. The charged Br ions significantly improve the carrier diffusion length^40^, resulting in negative charges moving across the interfacial energy barrier. Consequently, the secondary carriers are transferred to Ir(MDQ)_2_(acac) for red phosphorescent emission. For the same reason, the charged movable Br ions greatly facilitate magnetic field current, even under very subtle magnetic intensity with non-ionized polymer, which normally requires over hundreds of mT^41^. More discussion is presented in supplementary information.

**Fig. 2:**
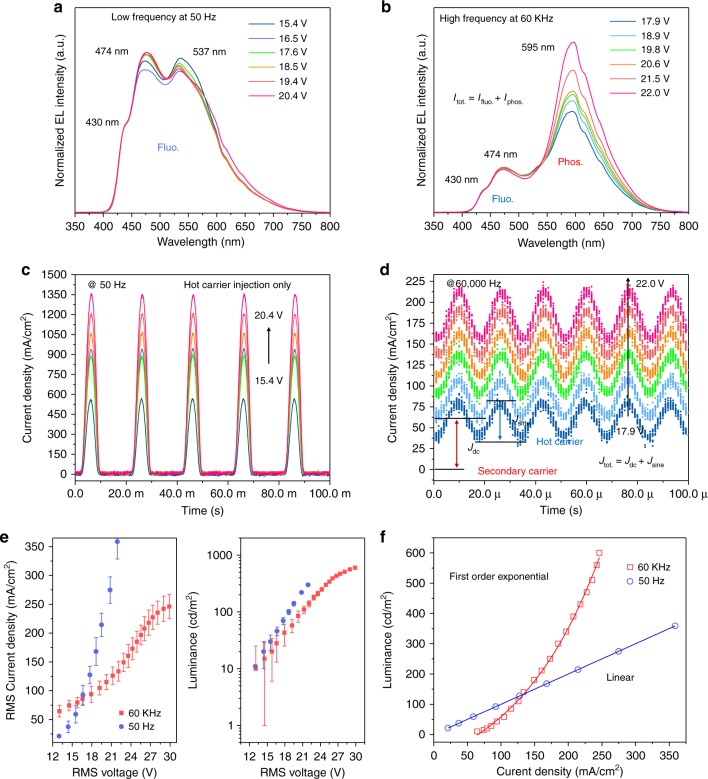
Electroluminescence (EL) spectrum and current characteristic transients with increasing RMS voltage under 50 and 60,000 Hz. **a** At 50 Hz, the electroluminescence (EL) spectrum with RMS voltage varying from 15.4 to 20.4 V, which correspond to luminances of 15–210 cd/m^2^. Hot carrier injection is the only current contribution. **b** At 60,000 Hz, the EL spectrum with RMS voltage varying from 17.9 to 22.0 V, which correspond to luminances of 40–240 cd/m^2^. **c** Time-resolved current at 50 Hz. Hot carrier injection is the only current contribution. **d** Time-resolved current at 60,000 Hz with various voltages (17.9–22.0 V). Secondary carriers (*J*_dc_) and hot injection carriers (*J*_sine_) have mutual contributions to the EL spectrum and current density characteristics. **e** *J*_rms_–*V*_rms_ and *L*–*V*_rms_ characteristics of AC-OEL devices at 50 and 60,000 Hz, respectively. **f** Luminance as a function of current density at 50 and 60 kHz

Figure 2a, b show the evolution of the device’s EL spectrum with increasing electric field at low and high frequencies. At the frequency of 50 Hz, there are trivial spectral shifts in Fig. 2a when the applied voltage varies from 15.4 to 20.4 V; however, no phosphorescence is observed. This is attributed to the dominant hot carrier injections, as shown in Fig. 2c. Electrons and holes are injected in the positive halves of voltage cycles while no current injection occurs in the reverse bias. Hot carrier injection is strongly related to the 474-nm-wavelength fluorescence.

For 60,000 Hz (secondary-carrier-related), as shown in Fig. 2b, the EL intensity of the fluorescent emission at 474 nm is nearly identical to that observed at 50 Hz. However, the 595 nm phosphorescent peak grows as the external electric field increases. Examining current transients in Fig. 2d, the total current density is the sum of the root-mean-square (RMS) values of the sinusoidal waveform and the direct current (DC) offset (*J*_tot_ = *J*_dc_ + *J*_sine_; the method used for current component determination can be found in Supporting Information). Under the lower voltage of 17.9 V, the DC current density and RMS sine current density are 60.2 and 36.6 mA/cm^2^, respectively. Compared to higher voltages (18.9, 19.8, 20.6, 21.5, and 22.0 V), the DC current densities are dramatically increased to 82.3, 114.9, 138.5, 164.1, and 184.6 mA/cm^2^ while relatively constant RMS sinusoidal currents of 37.8, 39.4, 42.1, 43.7, and 45.2 mA/cm^2^, respectively, are observed. We suggest that the tremendous enhancement of the secondary charge current produces the growth of the 595-nm-wavelength phosphorescence.

In addition, *J*_rms_–*L*–*V*_rms_ characteristics at low frequency (50 Hz) and high frequency (60,000 Hz) are shown in Fig. 2e, in which the maximum brightnesses, namely, 360 cd/m^2^ in blue and 600 cd/m^2^ in red, are of the order that is necessary for devices for personal display use. Figure 2f shows the relation between *J*_rms_ and *L* at the frequencies of 50 Hz and 60 kHz. The 50 Hz curve is fitted by a linear function, which has been widely demonstrated in the standard DC-driven organic light-emitting diodes (OLEDs). The linear fit strongly suggests that the exciton concentration inside the device remains below the level at which multi-exciton effects are dominant, such as Augur, quenching, and dissociation. In contrast, the 60 kHz curve can be fitted not by a linear function but by a first-order exponential function, which indicates an improved exciton recombination efficiency, which rules out the Augur recombination and quenching effects. The exponential line fits the data in both the high- and low-current regimes, which suggests that the phosphorescent exciton recombination efficiency is independent of the exciton density. These triplets are again generated by the free carriers from the e–h dissociation promoted by the AC-magnetic-field-assisted ISC suppression. Therefore, rather than the injection efficiency, the AC field is the main factor that impedes the singlet-to-triplet ISC. In consequence, a higher power efficiency is achieved at 60,000 Hz (1.5 lm/W; details are shown in Figure S6).

**Fig. 3:**
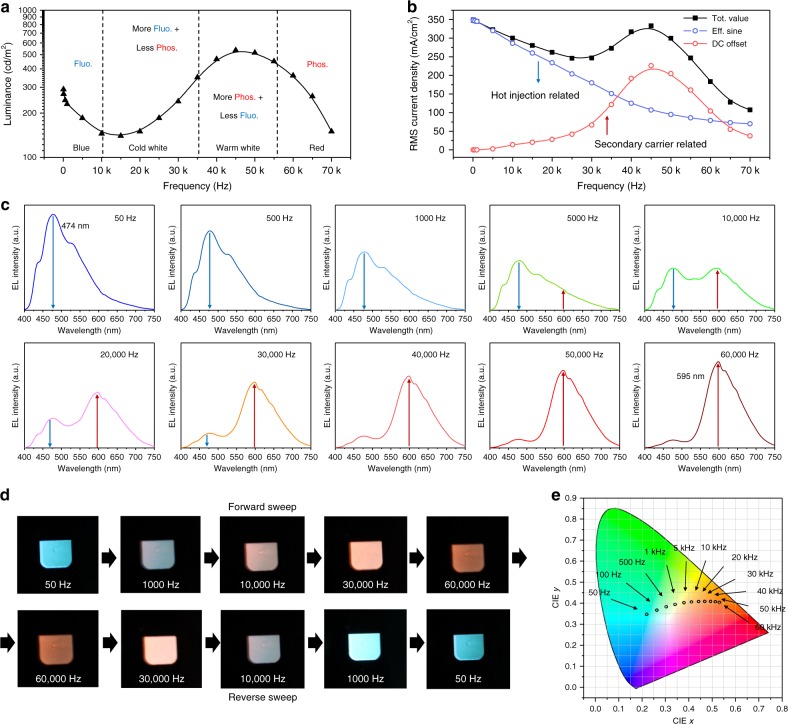
Color tunability of AC-OEL devices. **a** Plots of luminance as a function of frequency. **b** Current–frequency characteristics of AC-OEL devices and effective current analysis. **c** Electroluminescence (EL) spectral shifts of AC-OEL devices with driving frequencies of 50; 100; 500; 1000; 10,000; 20,000; 30,000; 40,000; 50,000; and 60,000 Hz. **d** Images of the color-tuning emission in the forward and reverse frequency sweeps. **e** CIE 1931 *x*–*y* chromaticity diagram, which shows the color shift from (0.23, 0.34) to (0.53, 0.40) with frequency variation between 50 and 60,000 Hz

The luminance–frequency characteristics of the color-tunable AC-OEL device are shown in Fig. 3a. The total emission of the device shifts between the fluorescent and phosphorescent contributions, whereas the luminance is relatively stable over the frequency. Corresponding to the F–P shift, the frequency characteristics are also shown with respect to current density in Fig. 3b. Analyzing the low-frequency regime (<1000 Hz) first, low frequencies lead to dominant blue fluorescence since, as we suspected, the device under low-frequency driving acts more like a carrier-injection-type diode in the forward and reverse biases (Figure S7).

At higher frequencies (>10,000 Hz), the current density consists of a sinusoidal contribution and a DC offset (see Figure S7), essentially reflecting both the displacement of the direct current injection and the secondary charge current, respectively. In Fig. 3b, the DC offset component of the current through the device starts at a very low level (13.8 mA/cm^2^) at 10,000 Hz and increases to 226.1 mA/cm^2^ at 45,000 Hz. In contrast, the RMS value of the sinusoidal component of the current waveform drops from 286.1 mA/cm^2^ at 10,000 Hz to 106.8 mA/cm^2^ at 45,000 Hz, which suggests a significantly reduced contribution of hot carrier injection to the total current at high frequency. These opposite trends illustrate that an electric field of >20,000 Hz applied to the capacitive device is sufficient to generate a magnetic field that is strong enough to yield secondary charge diffusion. The stronger AC magnetic field (due to higher frequency) suppresses ISC between singlet-state and triplet-state e–h pairs in the PFN-Br, thereby resulting in population enhancement of singlet e–h pairs at the F–P interface. The elevated singlet-triplet ratio promotes the generation of secondary charge carriers. The hopping transport of secondary electrons and holes in the organic semiconductor is acutely tied to the generation of radical triplet excitons in Ir(MDQ)_2_(acac), leading to red phosphorescence (as indicated in Fig. 3a). The coupling of the driver to the capacitive device has been taken into consideration by comparing the dominant capacitance of the device (1–3 nF) with the parallel external capacitance (530 pF) in the driver. Driving frequencies of >50,000 Hz cause insufficient carrier injection, resulting in the decrease in the number of e–h pairs and the total current reduction in Fig. 3b.

In Fig. 3c, the EL spectrum of the AC-OEL device shows a dramatic color change when the driving field frequency varies from 50; 100; 500; 1000; 10,000; 20,000; 30,000; 40,000; 50,000 Hz, up to 60,000 Hz. The 474 nm emission band is dominant at the low frequency of 50 Hz. As the driving frequency increases, the peaks at 430, 474, and 531 nm (due to PFN-Br’s fluorescent emission) are weakened; in contrast, the 595 nm peak (which corresponds to Ir(MDQ)_2_(acac)’s phosphorescent emission) grows rapidly and becomes the dominant emission band. All spectra are measured under 100 cd/m^2^, integrated for 500 ms and averaged over 5 runs. The operating pixel images in Fig. 3d provide us with a clearer picture of the color change with frequency in the forward and reverse sweeps. As indicated by the marked circles, the CIE coordinates in Fig. 3e start from (0.23, 0.34) at 50 Hz, cross the white zone, and reach (0.53, 0.40) in the red zone at 60,000 Hz. Another graphic display of the AC-OEL pixel color change with driving frequency is shown in Movie S2. We also observed non-homogenous emission from the pixel, which is mainly attributed to two major mechanisms: the non-homogenous thin film of PFN-Br and the uniformity of the AC magnetic field coupling. The thin PFN-Br layer at the substrate edge has an e–h generation zone close to the F–P interface, which facilitates the drift of the secondary charges, resulting in red emission. The AC field coupling is also spatially dependent, as shown in Fig. 1f, which suggests a stronger magnetic field at the substrate edge than at the center. Therefore, the spatially dependent magnetic field also leads to the red color shift that first occurs in the pixel area near the substrate edge. Further analysis of the non-homogenous emission can be found in Supplemental Information.

**Fig. 4:**
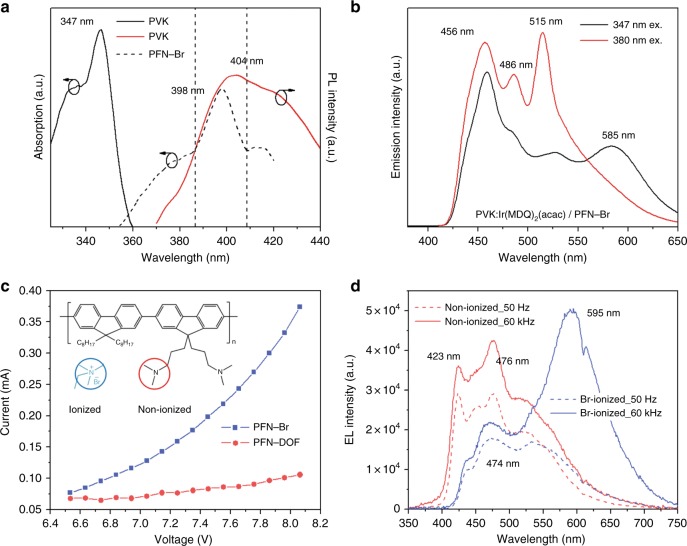
Photoluminescence (PL) spectroscopy for organic light emitting materials and energy transfer analysis. **a** Excitation and emission spectra for PVK film and PFN-Br film on glass. **b** PL spectra for glass/PVK:3 wt% Ir(MDQ)_2_(acac)/PFN-Br with two excitation wavelengths : 347 nm and 380 nm. **c** Current–voltage characteristics of PFN-Br (ionized) and PFN-DOF (non-ionized). **d** EL spectral shifts of PFN-Br (ionized) and PFN-DOF (non-ionized) at 50 Hz (magnetic-free) and 60,000 Hz (magnetic-engaged)

To further study the energy transition at the F–P interface, photoluminescence (PL) spectroscopy is performed, and the results are shown in Fig. 4a, b. The absorption of PFN-Br (peaks at 398 nm) shows a large overlap with the PL spectra of the host PVK (which show a wide peak at 404 nm) in Fig. 4a. This implies an efficient Fӧrster energy transfer route between PVK and PFN-Br (Fӧrster resonance energy transfer efficiency ~21.1%, calculated from Figure S8). In the PL spectra for PVK:3 wt% Ir(MDQ)_2_(acac)/PFN-Br shown in Fig. 4b, an extra 585 nm peak due to Ir(MDQ)_2_(acac) was detected when the sample was excited by 347 nm (PVK’s strongest absorption); in contrast, under 380 nm excitation (PFN-Br’s strongest absorption), this 585 nm peak is absent. This suggests that direct energy transfer between PFN-Br and Ir(MDQ)_2_(acac) is not allowed. The Fӧrster energy transfer from PVK to PFN-Br and the forbidden transfer between PFN-Br and Ir(MDQ)_2_(acac) explains the blue florescence that is due to PFN-Br in hot carrier injection.

Because of the full ionization of poly[(9,9-bis(3’-(N,N-dimethylamino)propyl)-2,7-fluorene)-alt-2,7-(9,9-dioctylfluorene)] (PFN-DOF) by Br atoms, PFN-Br possesses many moveable negative charges among the main polymers. Figure 4c shows the electron mobility enhancement by the diffusive Br-negative ions compared with PFN-DOF. The ionic conductivity is the most important property of solid polymer electrolyte. By introducing the Br ions into the PFN to form an electrolyte system, the magnitude of the conductivity will be enhanced. The conductivity of PFN-Br (~7.8 × 10^−^^7^ S/cm) is significantly improved compared to that of PFN-DOF (~3.1 × 10^−9^ S/cm), which is due to the Br-anion movements under the electric field. Because of the long-distance diffusion, the movable Br ions are able to populate the free secondary electrons, which leads to the dissociation of more excitons, thereby facilitating carrier diffusion to the phosphorescent emission layer. For comparison with PFN-Br devices, we studied the spectral shift in PFN-DOF devices between a magnetic-field-free device (50 Hz) and an AC-magnetic-field-coupled device (60,000 Hz) in the same architecture. In Fig. 4d, the fluorescence of PFN-DOF (at 423 and 476 nm) is promoted, which implies the promotion of singlet-state excitons by suppressing ISC of PFN-DOF. However, the color change is almost unnoticeable in PFN-DOF, which suggests that the secondary carriers are unable to reach the phosphorescent sites without the aid of Br ions. More work about the magnetic field effect on PFN-DOF can be found in supplementary information and Figure S9. In the absence of a PFN-Br layer, the excitons still can be formed and recombined at the p–n interface. However, the extremely low radiative recombination rate of TPBi facilitates exciton decay directly at the Ir(MDQ)_2_(acac) phosphorescent sites and the PVK fluorescent host, which was demonstrated by the EL spectra in Figure S10.

## Discussion

**Fig. 5:**
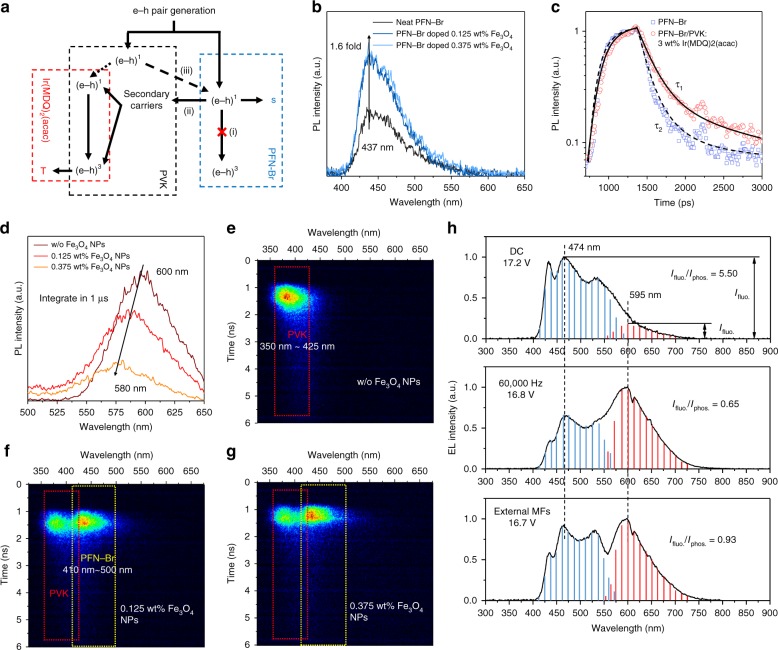
Energy transfer analysis for the fluorescence–phosphorescence (F–P) emission unit in the presence of magnetic field effect. **a** Schematic diagram of e–h pair excited energy transfer mechanisms among PVK (host), Ir(MDQ)_2_(acac) (phosphorescent dopant), and PFN-Br (fluorescent material) with magnetic-suppressive ISC. (e–h)^1^ and (e–h)^3^ represent singlet and triplet intermolecular e–h pairs. S and T are singlet and triplet excitons. **b** Time-integrated PL spectra that were measured at 437 nm for pure PFN-Br thin film, PFN-Br doping 0.125 wt% Fe_3_O_4_ NP film, and PFN-Br doping 0.375 wt% Fe_3_O_4_ NP film after constant excitation at 280 nm (10 Hz, 500 fs, and 130 μJ/cm^2^). **c** Time-resolved PL decay transients at 410–500 nm integration. **d** Time-integrated PL spectra (>1 μs) for glass/PVK:3 wt% Ir(MDQ)_2_(acac)/PFN-Br, glass/PVK:3 wt% Ir(MDQ)_2_(acac)/PFN-Br:0.125 wt% Fe_3_O_4_, and glass/PVK:3 wt% Ir(MDQ)_2_(acac)/PFN-Br:0.375 wt% Fe_3_O_4_. The streak images for glass/PVK:3 wt% Ir(MDQ)_2_(acac)/PFN-Br (**e**), glass/PVK:3 wt% Ir(MDQ)_2_(acac)/PFN-Br:0.125 wt% Fe_3_O_4_ (**f**), and glass/PVK:3 wt% Ir(MDQ)_2_(acac)/PFN-Br:0.375 wt% Fe_3_O_4_ (**g**) for 6 ns. **h** EL spectra with no magnetic field, internal AC magnetic field, and external magnetic field due to Fe_3_O_4_ NPs

Figure 5a shows the overall energy transfer at the PVK:Ir(MDQ)_2_(acac)/PFN-Br heterojunction. There are three processes that must be addressed:

1. ISC of e–h pairs in PFN-Br is magnetic-field-sensitive. In the presence of a magnetic field, energetically inaccessible T− and T+ states yield a redistribution of singlet and triplet excited states (S_0_:T_0_ = 1/2:1/2) in PFN-Br, as has been predicted theoretically^41–46^,
2. Accumulated singlet-spin e–h pairs are dissociated into diffusive secondary carriers with the assistance of Br ions;
3. The energy transfer of e–h pairs between PVK and PFN-Br is efficient but negligible because of the insufficient number of singlet-state excitons in PVK.

The AC magnetic field is only present in the high-frequency electric field. The magnetic field influences the ratio of singlet and triplet spin excitons at the p–n junction where e–h pairs are generated from the injected carriers. As a consequence, the 1:3 singlet–triplet ratio is violated and the singlet spin exciton accumulation occurs in the fluorescent emission layer. In contrast, it is believed that the exciton generation at the p–n interface would not be affected by the AC field due to the constant exciton generation zone.

An instructive and elegant confirmation of the magnetic field effects on F–P tunable emission can be obtained through the use of Fe_3_O_4_ magnetic NPs as a magnetic field alternative. The nano-magnetite-induced magnetic field is extremely local; hence, it will not introduce extra, unpredicted magnetic field effects as a uniform external magnetic field may do (for instance, possible magnetic field effects on the PVK). Moreover, it has been reported that magnetic field gradients due to Fe_3_O_4_ nanocrystals are more effective in suppressing ISC processes than static external fields for DC devices^1,42^. The ferromagnetic Fe_3_O_4_ magnetic NPs used here have an average diameter of 30 nm (Figure S11), which has been demonstrated by the X-ray diffraction (XRD) pattern (Figure S12), and are widely accepted as a ferromagnetic material since superparamagnetic behavior only exists in Fe_3_O_4_ magnetic NPs with diameters of <10 nm^47–49^.

The 1.6-fold magnetic-field-enhanced singlet-state excitons in PFN-Br were directly observed in time-integrated PL spectra in Fig. 5b, while 0.125 wt% Fe_3_O_4_ NPs was doped in PFN-Br. A slight PL intensity increase is observed in 0.375 wt% Fe_3_O_4_ NP-doped PFN-Br, indicating the 50% theoretical upper limit of singlet excitons. A constant excitation was used in the experiment: *λ*_ex._ = 280 nm. In Fig. 5c, we show the picosecond-transient PL intensity of PVK:Ir(MDQ)_2_(acac)/PFN-Br (*τ*_1_ = 319.7 ps, where *τ*_0_ is the time required for the PL to fall to 1/*e* of the initial intensity) and PFN-Br (*τ*_2_ = 171.7 ps) for 410–500 nm emission (which corresponds to PFN-Br’s fluorescence). We attribute the PL radiative delay to the short exciton diffusion in PFN-Br, which has a negligible impact on Ir(MDQ)_2_(acac)’s phosphorescence. Furthermore, the relatively reduced PL intensity of Ir(MDQ)_2_(acac)’s phosphorescent emission (580 nm–600 nm) in Fig. 5d provides evidence that no diffusive exciton is involved in color tunability even though singlet-state excitons are saturated with doping magnetic NPs in PFN-Br. Therefore, the possibility of diffusive excitons crossing the interface is excluded; in contrast, Br-ion-enhanced secondary carriers are demonstrated. In addition, we analyzed the streak image for the sample of PVK:3 wt% Ir(MDQ)_2_(acac)/PFN-Br in Fig. 5e. No emission is observed except for an intense emission band (350–425 nm), which corresponds to PVK’s fluorescence. However, PFN-Br’s fluorescent emission (410–500 nm) becomes observable when 0.125 wt% Fe_3_O_4_ (Fig. 5f) or 0.375 wt% Fe_3_O_4_ (Fig. 5g) are blended into PFN-Br. This result demonstrates that massive numbers of radical singlet-spin pairs are formed in PFN-Br due to the influence of the magnetic field on the F–P emitting unit. Ir(MDQ)_2_(acac)’s phosphorescent emission cannot be captured in the first several nanoseconds in either case because of long-lived triplet excitons but appears at 600 nm in time-integrated spectra, as shown in Figure S13.

To confirm the importance of the magnetic effect on the F–P emitting unit, we measured the EL spectra of analogous devices (the device structures are described in detail in Figure S14) in Fig. 5h with zero magnetic field coupling (DC driving), internal AC magnetic field (60,000-Hz driving), and external NP magnetic field (DC driving). A large F–P intensity ratio (*I*_f_/*I*_p_) of 5.5 was observed when the magnetic field was eliminated. In contrast, the F–P intensity ratios are 0.65 and 0.93 in the AC magnetic field and Fe_3_O_4_ NC magnetic field, respectively. This implies that the low F–P intensity ratio is evidence that secondary charges, which originate from singlet e–h pair accumulation, are able to facilitate the generation of triplet excitons in the Ir(MDQ)_2_(acac) molecules.

In summary, the application of internally generated AC magnetic fields in a unique AC-OEL architecture is demonstrated to be an efficient method for manipulating the ratio of singlet- and triplet-spin pairs. The magnetically sensitive ISC can modify the probability of singlet-spin e–h pairs in organics (PFN-Br), which significantly populates diffusive secondary charges to achieve phosphorescence emission (Ir(MDQ)_2_(acac)). By varying AC magnetic fields that are coupled at an F–P heterointerface in the AC-OEL device, the emission can be shifted between blue fluorescence (singlet-spin-related) and red phosphorescence (triplet-spin-related) with no significant brightness changes. Conventional contributions to color shifts that are associated with spatial shifting of recombination zones or exciton redistribution and quenching under high voltage have been excluded by our experiments. While the unusual properties of magnetically coupled AC-OEL devices may lead to other breakthrough devices, our work opens new doors to a more detailed understanding of radical-pair manipulation in the quantum electrodynamics and spintronics of organic materials.

## Materials and methods

### Materials and device fabrication

The AC-OEL devices were built on a 2.54 × 2.54 cm^2^ glass substrate precoated with a 140-nm-thick layer of ITO with a sheet resistance of ~10 ohms per square. The ITO glass substrate was cleaned in an ultrasonic bath with acetone followed by methanol and isopropanol for 1 h each and dried in vaccum oven for 2 hours. Before spin-coating, the ITO substrates were exposed in UV-ozone for 30 min. To efficiently control the carrier transport under AC driving, PEDOT:PSS doped with 18 wt% ZnO NPs (~35 nm) was spun onto the substrate to form a gate-and-hole-generation layer. For the dual-emission-layer unit, a layer of PVK with 3 wt% Ir(MDQ)_2_(acac) was spin-coated in chlorobenzene at a concentration of 10 mg/mL at 2000 rpm, followed by baking at 100 °C for 30 min. The second emission layer was obtained by spin coating the 5 mg/mL (20 nm), 8 mg/mL (50 nm), or 10 mg/mL (70 nm) PFN-Br blends in methanol at 3000 rpm and dried at 95 °C for 20 min. Iron oxide magnetic nanopowder (Fe_3_O_4_ NPs ~30 nm) was purchased from Sigma-Aldrich and was pre-functionalized by *N*-succinimidyl ester for easy dispersion in a suitable solvent. A 40 nm electron-transport material (TPBi) and a 150 nm top Al electrode were deposited by thermal evaporation through a shadow mask with a 0.16 cm^2^ opening at the rates of 0.2 and 2 Å/s, respectively. Before testing, the devices were sealed by quartz caps with UV curing adhesive in nitrogen atmosphere in a glove box.

### Device EL and PL measurement

The AC-OEL devices were measured in ambient air at atmospheric pressure and room temperature (25 °C) with encapsulation. A 200 MHz function/arbitrary waveform generator (Agilent 33220A) connected to an amplifier (Trek PZD700A M/S) provided a sinusoidal signal with suitable voltage and frequency. A power analyzer (Zimmer LMG95) was utilized to read the RMS values of voltage and current on the AC-OEL devices. At the same time, voltage waveforms and current waveforms were recorded by a Tektronix TPS 2024B oscilloscope to validate the voltage and current values. The method that we used to determine the AC and DC current components is described in detail in Supplemental Information. A photometer (ILT 1400-A) was used to measure the out-coupling luminance. The entire system was connected and controlled by a computer. To obtain accurate and reliable measurements of luminance, each turn-on measurement of the pixels was integrated over 2000 ms and averaged 5 times instead of fast sweeping to yield good-looking curves. The EL spectra for color-tunable AC-OEL devices were collected by an ILT 950 spectroradiometer (International Light Technologies) while the driving frequency was varied easily by a function waveform generator. The PL spectra for PVK, PFN-Br, and PVK:Ir(MDQ)_2_(acac)/PFN-Br thin films were measured by a fluorescence spectrometer (PerkinElmer LS50B).

### Streak camera test

For measurement of the transient PL characteristics, short-pulse excitation with a pulse width of 200 fs and a wavelength of 280 nm was used in combination with a Hamamatsu C2830 streak camera system that mainly consisted of a C2830 streak camera and an M2547 fast sweep unit (resolution <10 ps). F–P lifetime measurements were carried out via a photodiode with UV laser illumination in dark-room ambient at 25 °C.

###  Transmission electron microscopy (TEM) and atomic force microscopy (AFM) images

A TEM (JEOL JEM-1200EX) was utilized to analyze the morphology of Fe_3_O_4_ NPs dispersed in PFN-Br methanol solution. The surface morphology analyses of PVK and PFN-Br were performed using an AFM (Asylum Research MFP-3D-BIO).

### X-ray diffraction (XRD) pattern

The crystalline phase analysis was performed using an X-ray powder diffractometer (Bruker D2 PHASER) in ambient. The result was averaged over 10 measurements.

## Electronic supplementary material

Research summary

Moive1

Moive2
