# Supplementary material for: Tailoring spin mixtures by ion-enhanced Maxwell magnetic coupling in color-tunable organic electroluminescent devices
Source: Light Sci Appl. 2018 Aug 1;7:46. doi: 10.1038/s41377-018-0046-5 (PMC6107016; doi:10.1038/s41377-018-0046-5)
Supplement: Supplementary file 1 — Research summary [file 41377_2018_46_MOESM1_ESM.docx]

Supplementary Materials

Title

- Tailoring Spin Mixtures by Ion-Enhanced Maxwell Magnetic Coupling in Color-Tunable Organic Electroluminescent Devices.

**Authors**

Junwei Xu,^1,2^ Yue Cui^1,3^, Gregory M. Smith,^1,2^† Peiyun Li,^2,4^ Chaochao Dun,^1,2^ Linqi Shao,^1,5^ Yang Guo,^1,6,7^ Hongzhi Wang. ^6,7^ Yonghua Chen^8^, David L. Carroll,^1,2^*

**Affiliations**

(1) Center for Nanotechnology and Molecular Materials, Wake Forest University, Winston-Salem, NC 27109, USA.

(2) Department of Physics, Wake Forest University, Winston-Salem, NC 27106, USA.

(3) Key Laboratory of Luminescence and Optical Information (Ministry of Education), Institute of Optoelectronics Technology, Beijing Jiaotong University, Beijing 100044, P.R. China.

(4) Department of Electro-Optics, School of Engineering, University of Dayton, Dayton, OH 45469, USA.

(5) Department of Mathematics, Wake Forest University, Winston-Salem, NC 27106, USA.

(6) State Key Laboratory for Modification of Chemical Fibers and Polymer Materials, Shanghai 201620, P.R. China.

(7) College of Materials Science and Engineering, Donghua University, Shanghai 201620, P.R. China.

(8) Institute of Advanced Materials, Nanjing Tech University, Nanjing, Jiangsu 211816, P.R. China

† Present address: Department of Physics and Astronomy, College of Charleston, `Charleston, SC 29424, USA.

* Corresponding Author: [carroldl@wfu.edu](mailto:carroldl@wfu.edu)

**This file contains:**

Supplementary text

Figure S1 | Spin pair remixing in magnetic field.

Figure S2 | Atomic force microscopy images of PVK and PFN:Br

Figure S3 | Numerical simulation on dynamic magnetic fields at the exciton generation interface with frequency variation.

Figure S4 | Blue and red pixels driven at 50 Hz and 60,000 Hz.

Figure S5 | Electroluminescence spectrum with PVK: 3 wt% FIrpic/PFN:DOF: 3 wt% Ir(MDQ)_2_(acac).

Figure S6 | Power efficiency of AC-OEL device at 50 Hz and 60,000 Hz.

Figure S7 | Current and voltage waveforms at 50 Hz and 60,000 Hz.

Figure S8 | PL spectroscopy of PVK: Ir(MDQ)_2_(acac)/PFN-Br and resonance energy transfer (FRET) efficiency.

Figure S9 | EL spectrum of PFN-DOF (non-ionized) with Fe_3_O_4_ NPs.

Figure S10 | EL spectrum for PVK with different doping ratios of Ir(MDQ)_2_(acac).

Figure S11 | TEM image of Fe_3_O_4_ NPs.

Figure S12 | X-ray diffraction pattern of Fe_3_O_4_ NPs.

Figure S13 | Time integrated streak images for magnetic NPs doped F-P emission unit.

Figure S14 | Device configurations of devices with interfacial AC magnetic field and Fe_3_O_4_ NP magnetic field.

Figure S15 | Energy level diagram of AC-OEL device.

Figure S16 | Chemical structures of PFN-Br and PFN-DOF in comparison.

Figure S17 | EL spectrum of device without and with magnetic fields (static or AC) in a constant current density of 100mA/cm^2^.

Figure S18 | EL spectrum of AC-OEL devices with a variety of Fe_3_O_4_ NPs concentrations.

Figure S19 | EL spectrum with different thicknesses of PFN-Br.

Figure S20 | F-P intensity ratio vs various thicknesses of PFN-Br.

Figure S21 | UV photoluminescence images of Ir(MDQ)_2_(acac) and PFN-Br in solutions.

Figure S22 | Red color distribution over the distance from substrate center.

Table S1. Materials used in this work.

Movie S1. Self-generated magnetic field dynamics.

Movie S2. Color change demonstration (blue to red).

**Operating mechanism of AC-OEL devices:**

The energy schematic of color tunable AC-OEL devices is shown in **Figure S15**. A 80nm layer of PEDOT:PSS doped 18 wt% ZnO nanoparticles (diameter~35 nm) was used as hole generation layer (HGL) as well as carrier gate. The gate layer is source of holes developed by hot carrier injection and polarization current within device. The electron-hole pairs generated by high frequency electric field dissociate to free carriers for exciton contribution. The polarization current is proportional to the time derivative of the electric field. In the positive and negative halves of AC cycles, ZnO gate has a significant contribution on carriers manipulation, especially at the interface of ITO and HGL. In the forward bias, the electrons remaining in HGL are extracted from device through ZnO gate. In the reversed bias, the energy barrier at ITO/ZnO interface trends to accumulate holes until these excessive mobile charges are neutralized in next cycle.

On the other hand, electrons are easily injected form Al electrode over TPBi (electron transporting layer). Both of holes and electrons are transported to PVK:Ir(MDQ)_2_(acac)/PFN-Br which is a fluorescence-phosphorescence hybrid emission layers. The excited hole and excited electron recombination occurs at the interface of PVK:Ir(MDQ)_2_(acac) and PFN-Br. This is because PFN-Br is a high performance electron transporting material as well.

In the DC driving mode, hot carrier injection is defined as a fact that an electron or a hole gains sufficient kinetic energy to overcome a potential barrier necessary to break an interface between layers. For negative charges, electrons are injected from Al electrode to F-P interface over TPBi (electron transporting layer). The electron injection energy can be estimated to the sum of the energy barriers of Al/TPBi (1.36 eV) and TPBi/PFN-Br (0.61 eV). In terms of positive charges, holes are generated at HGL then transferred to the F-P interface by overcoming a 0.2 eV energy barrier.

**Device power efficiency**

The power efficiencies of the device with and without AC magnetic field are plotted in the function of luminance in **Figure S6**. As we can see, the device at 50Hz shows a reduced power efficiency as luminance goes up due to the power loss at the interfacial barriers, which is quite normal in a regular OLED device. However, an efficiency enhancement was observed at higher luminance (corresponding higher voltage) in the 60kHz driving condition. This is because a stronger magnetic field is generated at higher voltage (brighter device) which facilitates the single exciton dissociation in PFN-Br compensating the power efficiency by the triplet exciton harvest in Ir(MDQ)_2_(acac).

**Mathematic derivation of varying electric and magnetic field:**

The magnetic fields in capacitor-based devices have to satisfy the Maxwell equations:

$\nabla\times E=-\mu\frac{\partial H}{\partial t}$ (1)

$\nabla\times H=\varepsilon\frac{\partial E}{\partial t}$ (2)

$\nabla\cdot\varepsilon_{0}E=0$ (3)

$\nabla\cdot\mu_{0}E=0$ (4)

Considering the boundary conditions,

$n\times E=0$ (5)

$n\cdot\varepsilon E=\eta_{f}$ (6)

$n\times H=k_{f}$ (7)

$n\cdot\mu H=0$ (8)

where $\eta_{f}$ is free charge plane density, $k_{f}$ is free charge plane current density.

$E=\{E_{r}\left( r,t \right), E_{\varphi}\left( r,t \right),E_{z}\left( r,t \right)\}$ (9)

$H=\{H_{r}\left( r,t \right), H_{\varphi}\left( r,t \right),H_{z}\left( r,t \right)\}$ (10)

Due to $E_{\varphi}\left( r,t \right)=E_{z}\left( r,t \right)=0$ and $H_{r}\left( r,t \right)=H_{z}\left( r,t \right)=0$

$E=E_{z}\left( r,t \right)e_{z}$ (11)

$H=H_{\varphi}\left( r,t \right)e_{\varphi}$ (12)

Thus, we can simplify the Maxwell equations as

$\frac{\partial E_{z}}{\partial r}=\mu\frac{\partial H_{\varphi}}{\partial t}$ (13)

$\frac{1}{r}\frac{\partial(rH_{\varphi})}{\partial r}=\varepsilon\frac{\partial E_{z}}{\partial t}$ (14)

Rewirte the above equations into

$\frac{1}{r}\frac{\partial}{\partial r}\left[ r\frac{\partial E_{z}}{\partial r} \right]=\varepsilon\mu\frac{\partial^{2}E_{z}}{\partial t^{2}}$ (15)

$\frac{\partial}{\partial r}[\frac{1}{r}\frac{\partial}{\partial r}(rH_{\varphi})]=\varepsilon\mu\frac{\partial^{2}H_{\varphi}}{\partial t^{2}}$ (16)

with zero order Bessel function ($J_{0}(z)$) and first order Bessel function ($J_{1}(z)$), we can solve time-varying electric field and magnetic field as follow:

$E\propto-\frac{V_{0}}{d}J_{0}\left( \beta_{0}r \right)\cos\omega te_{z}$ (17)

$H\propto-\frac{V_{0}}{d\sqrt{\mu/\varepsilon}}J_{1}\left( \beta_{0}r \right)\sin\omega te_{\varphi}$ (18)

$B\propto-\frac{{\sqrt{\varepsilon\mu}V}_{0}}{d}J_{1}\left( \beta_{0}r \right)\sin\omega te_{\varphi}$ (19)

where ${\beta_{0}}^{2}=\omega^{2}\varepsilon\mu$.

**Electric field analysis:**

The determination of recombination zone of holes and electrons is necessary to be clarified so that recombination zone movement is not the key factor that causes the color tunability of AC-OEL devices. PFN-Br is high performance electron transporting polymer used in literature. ^1,2^ So is its derivative, PFN-DOF. The measurements of μ of PFN-Br and PFN-DOF are performed in electron-only device (ITO/TPBi/PFN-Br or PFN-DOF/TPBi/Al) in **Figure 4c**. The μ of PFN-Br and PFN-DOF are extracted by fitting the J-V plots, the current density is expressed as,

$J=\frac{9}{8}\varepsilon\mu_{0}\frac{V^{2}}{d^{3}}exp(0.89\beta\frac{\sqrt{V}}{\sqrt{d}})$ (20)

$\boldsymbol{\varepsilon}$ is the permittivity of organic layers, $\boldsymbol{\mu}_{\mathbf{0}}$ is the zero-field mobility, V is the applied DC bias, d is the thickness of PFN-Br or PFN-DOF, $\boldsymbol{\beta}$ is the field-activation factor. More details can be found in literature. The electron mobility of PFN-Br and PFN-DOF are estimated to 1.41×10^-7^ cm^2^V^-1^s^-1^ and 3.69×10^-7^ cm^2^V^-1^s^-1^ respectively which are comparable to the-art-of-state electron transporting materials [TmPyPb (7.0×10^-7^), TPBi (3.2×10^-7^), Bphen (2.2×10^-6^), and BCP (7.7×10^-8^)].

In order to exclude recombination zone movement from the reasons of color tunability, we fabricate the AC-OEL device with structure of ITO/PEDOT:PSS doped ZnO NPs /PVK: 3 wt% FIrpic /PFN-DOF: 3 wt% Ir(MDQ)_2_(acac)/TPBi/Al. The spectra of the device are shown in **Figure S5** with varying frequency (50 Hz, 1,000 Hz, 10,000 Hz, 30,000 Hz, 50,000 Hz). There is barely spectrum shift, which suggests there is no recombination zone movement in AC-OEL devices. The exciton generation zone is located in the n-type material side since holes have higher mobility than electrons. This means there are insufficient singlet excited state excitons generated in PVK, plus only few of these singlets would transfer to PFN-Br over p-n interfaces. Thus, very few of singlets in PVK are transferred to Ir(MDQ)_2_(acac) molecules, resulting in nearly unobservable 600nm emission at 50Hz.

**Quantum calculation of singlet probability in magnetic field:**

In a pair of spins system, we know the Hamiltonian $H=-{g\mu(B_{s1}\cdot S_{s1}+B_{s2}\cdot S_{s2})}/\hbar$ which satisfy the Schrӧdinger equation

$i\hbar\frac{\partial\varphi}{\partial t}=H\varphi$ (21)

where $S_{1}=\left( \frac{\hbar}{2} \right)\sigma$ and $S_{2}=\left( \frac{\hbar}{2} \right)\sigma$ are spin operators. The magnetic fields in Hamiltonian incorporates both hyperfine field and external field. Under the week interaction between two spins assumption, the probability of being a singlet state at time t is

$P_{singlet}\left( t \right)={[\cos\left( \frac{\omega_{s1}t}{2} \right)\cdot\cos\left( \frac{\omega_{s2}t}{2} \right)+(B_{s1}\cdot B_{s2}/B_{s1}\cdot B_{s2})\cdot\sin(\frac{\omega_{s1}t}{2})\cdot\sin(\frac{\omega_{s2}t}{2})]}^{2}$ (22)

with $\omega_{s1}=g\mu B_{s1}/\hbar$ and $\omega_{s2}=g\mu B_{s2}/\hbar$. For the average of singlet probability:

$\left\langle P_{singlet}\left( t \right) \right\rangle=\int P_{singlet}\left( t \right)\cdot P(B_{s1})\cdot P(B_{s2})$ (23)

Evaluate $\left\langle P_{singlet}\left( t \right) \right\rangle$ where magnetic fields are selected from independent 3D Gaussian distributions. Thus, the average probability of being a singlet state and triplet are

$\left\langle P_{singlet}\left( t \right) \right\rangle=\frac{(1+\chi+\chi^{2})}{3}$ (24)

$\left\langle P_{singlet}\left( t \right) \right\rangle=\frac{(2-\chi-\chi^{2})}{9}$ (25)

respectively, where $\chi$ is defined as $(1-\left( g\mu\sigma t/\hbar\right)^{2})\cdot exp[-\left( g\mu\sigma t/\hbar\right)^{2}/2]$. These average probabilities can provide us the quasi-steady-state singlet-triplet ratio without thermal equilibrium consideration. After long time reorientation through hyperfine field, the singlet-state probability of 25% is reached. A. Cohen evaluated the singlet-triplet ratio in more general case where magnetic fields are generated by ferromagnetic nanocrystals by Monte Carlo simulation.^3^ The quasi-steady-state singlet probability approaches 50% experimentally.^4^

**Calculate resonance energy transfer (FRET) efficiency**

The fluorescence absorption and emission are a sample with donor + acceptor and for an identical reference sample without acceptor, the FRET efficiency is given by

$$E=1-\frac{A_{D}}{A_{DA}}\times\frac{I_{DA}}{I_{D}}$$

where *A*_D_ and *A*_DA_ are the donor absorbances at the excitation wavelength in the donor-only and donor +acceptor samples, and *I*_DA_ and *I*_D_ are the total donor fluorescence intensities in presence and absence of acceptor, respectively The donor and acceptor are PVK and PFN-Br in current case. The absorbances and emissions of PVK and PVK:PFN-Br were tested at the same excitation wavelength (346 nm) and the same slit bandwidths (15 nm) are used for two emission measurements. The results are shown in **Figure S8**. The calculated FRET efficiency from PVK to PFN-Br is estimated to 21.1%.

**Secondary carriers without Br ion enhancement**

Compared to its sister polymer PFN-DOF, PFN-Br is fully ionized by Br ions as chemical structures shown in **Figure S16**. Because of long diffusive distance, Br ions facilitate the dissociation of excitons and populates the free secondary electrons to PVK layer. Thus, without the aid of ions in PFN-DOF, the 600 nm phosphorescent emission become weak and hard to record. Let us take a look at **Figure S9a** where we investigated the spectral shift between AC field free device (50 Hz) and AC field coupled device (60 kHz). As we expected, the color change is almost unnoticeable, however, the fluorescence of PFN-DOF increases implying the promoted singlets by suppressed ISC. Thus, the color change effect on PFN-DOF is significantly reduced compared with that on PFN-Br, which results from the absence of Br ions.

A considerable change in EL spectrum is observed as additional magnetic field from Fe_3_O_4_ NPs is applied on PFN-Br which shown in **Figure S9b**. Both 477 nm and 600 nm peaks are dramatically promoted while 0.06 wt% Fe_3_O_4_ NPs are doped into the PFN-DOF matrix. The ISC rate in PFN-DOF is sufficiently weaken by the AC magnetic field and the Fe_3_O_4_ NPs. The singlet-spin e-h pair accumulation becomes easier to reach at the expense of consuming triplet polaron pairs in PFN-DOF. The dissociation of singlet e-h pairs in large population causes diffusive secondary charge transfer from PFN-DOF to Ir(MDQ)_2_(acac) molecule sites. However, without the aid of Br ions, this transfer seems relative weak compared with in PFN-Br.

Similar scenarios of enhanced 600 nm peak and 477 nm emission are discovered in **Figure S9c** and **d** while higher concentrations of Fe_3_O_4_ NPs, 0.13 wt% and 0.26 wt%, involve in PFN-DOF polymer with 60 kHz magnetic field. 0.13 wt% doped device shows 20.1% and 112.5% increases of 477 nm emission and 600 nm peak, respectively. These increase rates drop to 9.7% (at 477 nm) and 58.5% (at 600 nm) in 0.26 wt% case.

Overall, in comparison between PFN-DOF and PFN-Br (Br ionized), the lack of Br ions significantly reduced the promotion of 600nm phosphorescent emission no matter in AC field or static magnetic field. This is due to the less diffusive secondary carriers are generated without Br ions. Thus, a relative large magnetic field effect is present in PFN-Br with a magnetic field less than 1 mT.

**Static external magnetic field experiment**

EL spectral shift under external magnetic fields on such devices would provide the fundamental validation of the Maxwell magnetic field effect. In reality, it is inevitable that the ISC processes of PFN-Br as well as PVK would be impeded, which leads us to a more complicated consequence. Therefore, a simplified device configuration is used which only has one host-guest emission unit. The host material is PFO which has a magnetic insensitive ISC. The Ir(MDQ)_2_(acac) dopants play the role of phosphorescent guest. By comparing the magnetic field effects between time-dependent self-generated magnetic field and external magnetic field, we are able to rule out other physical mechanisms underlying the magnetic related spectrum shift.

As shown in **Figure S17**, the emission peaks at 433 nm and 460 nm are corresponding to the PFO fluorescence, and 590 nm emission is phosphorescence due to triplet states of Ir(MDQ)_2_(acac). The ratio between fluorescent peak and phosphorescent peaks reveals the singlet-state and triplet-state spin populations. As we expected, a blue shift was observed when the device is driven under AC MFs or static MFs compared with no external MFs. The presence of external magnetic fields enable the enhancement of fluorescent peaks due to suppressing the ISC process of PFO. Therefore, the additional experimental data shows the verification that external field and internal field outcomes the same singlet-triplet tuning, which is the direct and clear evidence to Maxwell magnetic field effect in AC-OEL devices. These results are quite consistent with the magnetic EL effect that Prof. Hu reported a decade ago (*Nat. Mater.* **6,** 985–91, 2007). They may not directly relate to this report, but again we still add them to the supplemental information since we wish to confirm the basic magnetic EL effect on our devices.

**Non-uniform color shift**

The mechanism of the non-uniformity has not been fully understood at this moment. However, a possible reason is the saturation of triplet-spin excitons in Ir(MDQ)_2_(acac) sites due to the low doping ratio and the long decay of the phosphorescence. The similar phosphorescence saturation was observed in EL spectrum by increasing Fe_3_O_4_ NPs (0.06 wt%, 0.13 wt%. 0.26 wt%) as shown in **Figure S18**. Without magnetic NPs, there is almost no phosphorescent emission at 600 nm. The peak appears when only 0.06 wt% Fe_3_O_4_ NPs are added into fluorescent layer. The 600 nm peak gets stronger while the doping ratio increases to 0.13 wt%. However, more magnetic NPs (0.26 wt%) in the system do not continue to promote the phosphorescent emission. This might be due to the triplet-spin state of Ir(MDQ)_2_(acac) are saturated by the high concentration of secondary carriers under the intensive external magnetic field and low density of Ir(MDQ)_2_(acac) sites at the F-P interfaces (shallow carrier penetration depth due to the lack of diffusive ions).

**DC/AC current components analysis**

Step 1: Set Tektronix oscilloscope in DC coupling mode.

Step 2: Apply voltage on the device.

Step 3: The current waveforms of the devices were collected via the oscilloscope.

Step 4: Switch to AC coupling mode. Only the AC sinusoidal components of the waveforms were displayed and collected.

Step 5: Subtract the DC coupled waveform from AC coupled waveform. The left is defined as the DC current component of the devices.

**Additional references**

1. Huang, F., Wu, H., Wang, D., Yang, W. & Cao, Y. Novel Electroluminescent Conjugated Polyelectrolytes Based on Polyfluorene. *Chem. Mater.* **16,** 708–716 (2004).

2. Tian, Y., Xu, X., Wang, J., Yao, C. & Li, L. Solution-processed white organic light-emitting diodes with enhanced efficiency by using quaternary ammonium salt doped conjugated polyelectrolyte. *ACS Appl. Mater. Interfaces* **6,** 8631–8638 (2014).

3. Cohen, A. E. Nanomagnetic control of intersystem crossing. *J. Phys. Chem. A* **113,** 11084–92 (2009).

4. Wang, J., Chepelianskii, A., Gao, F. & Greenham, N. C. Control of exciton spin statistics through spin polarization in organic optoelectronic devices. *Nat. Commun.* **3,** 1191 (2012).

5. Reineke, S. *et al.* White organic light-emitting diodes with fluorescent tube efficiency. *Nature* **459,** 234–8 (2009).


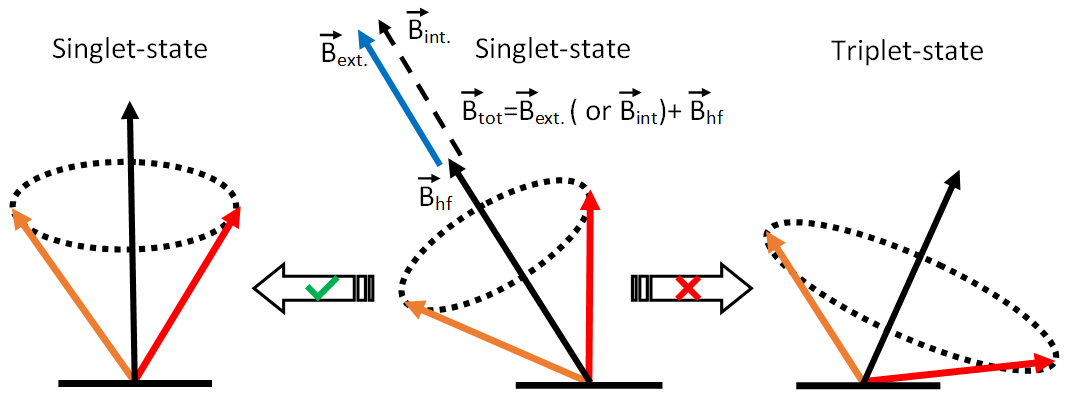


Figure S1 | Polaron pair spin remixing with external or internal magnetic field.


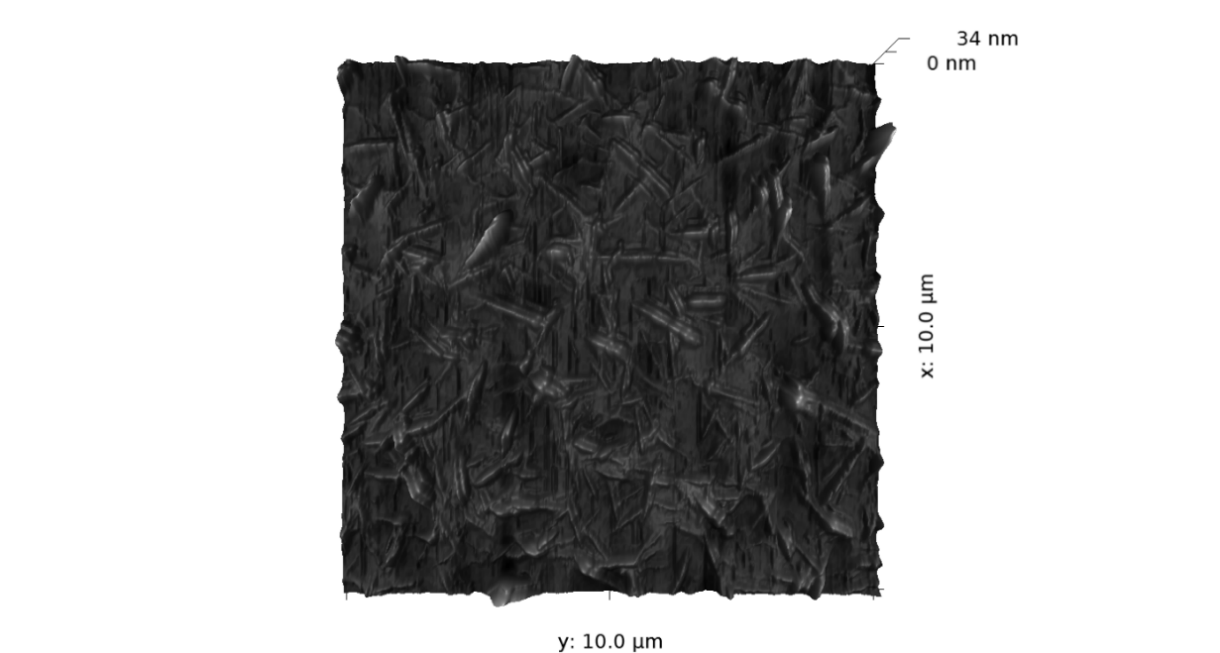


(a)


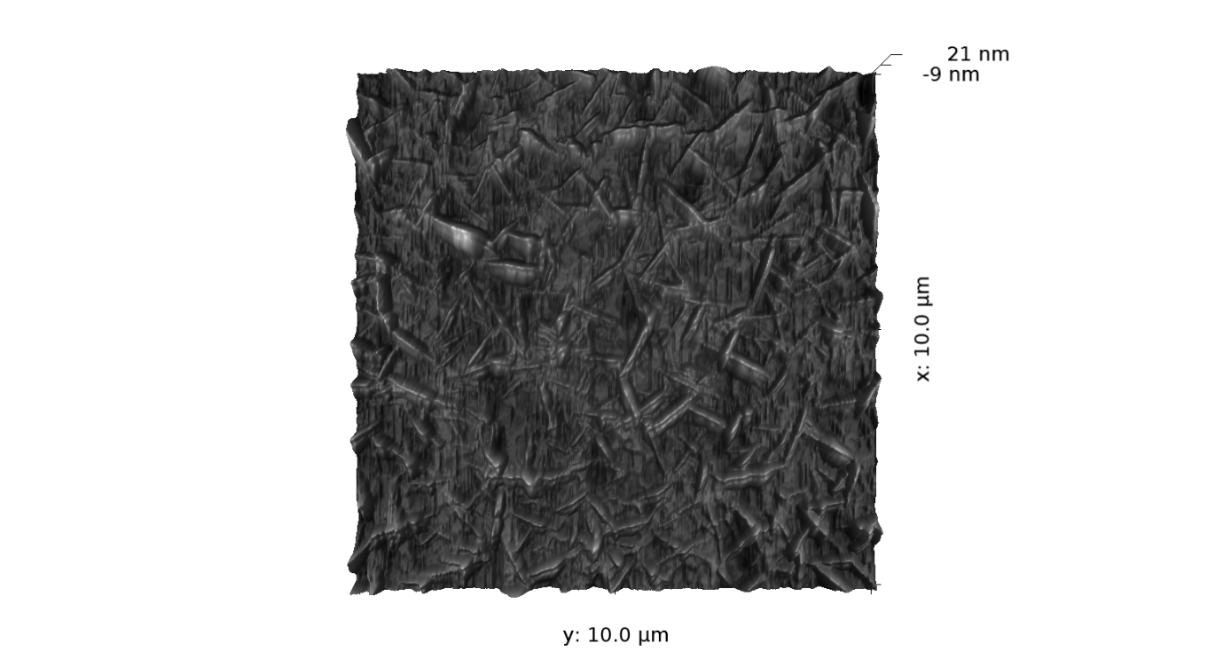


(b)


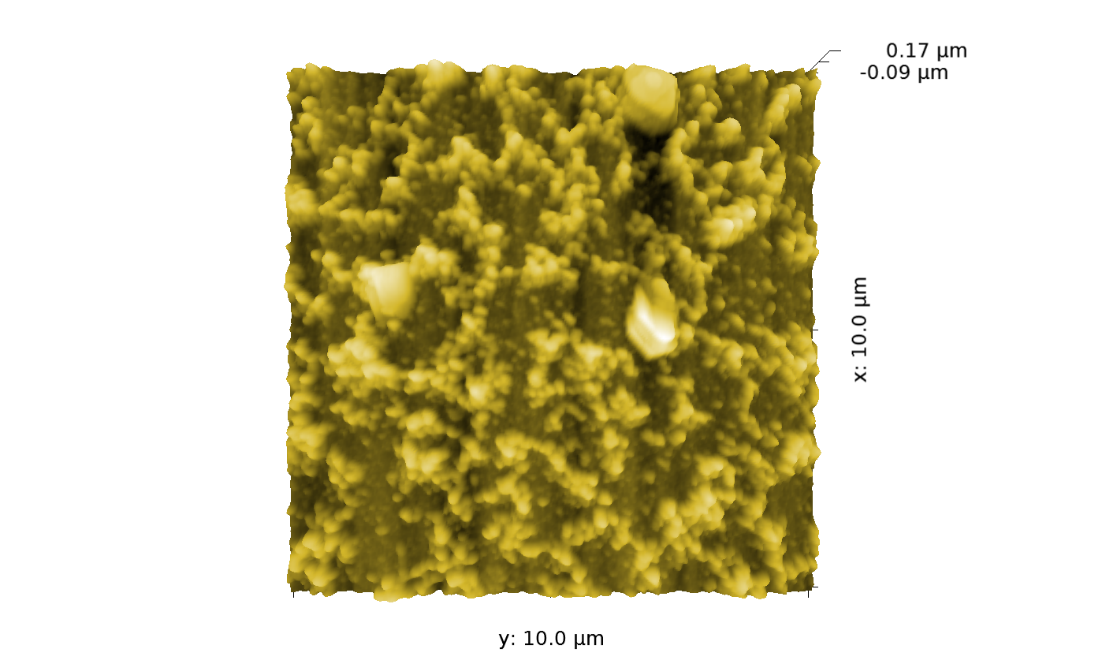


(c)


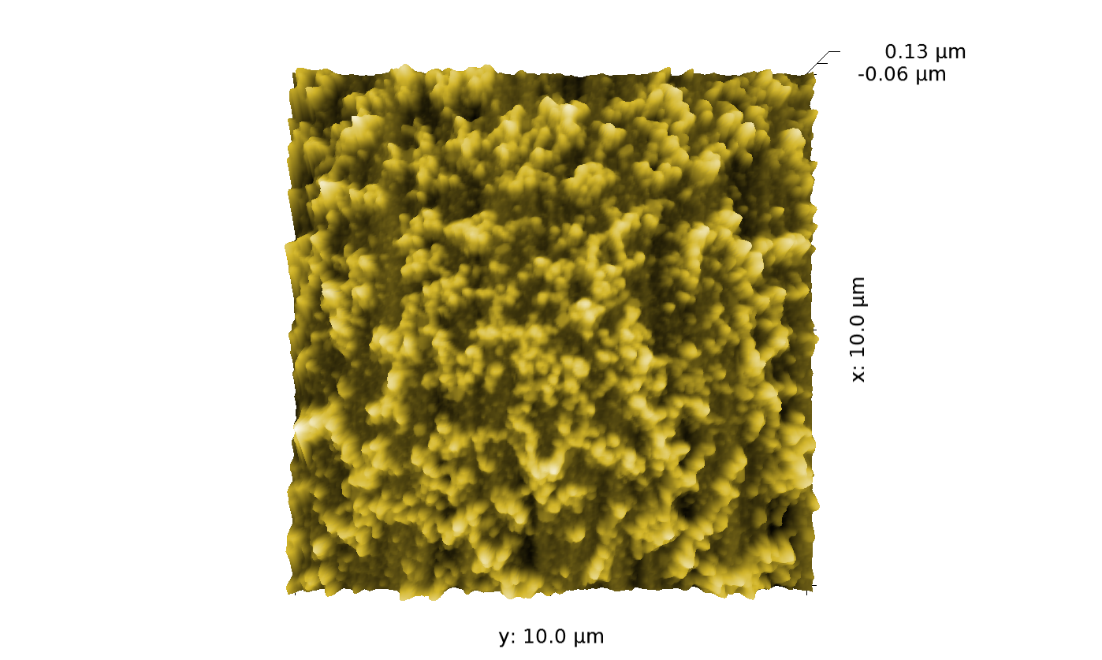


(d)

Figure S2 | AFM images of PVK:Ir(MDQ)_2_(acac) before annealing (a) and after 100 degrees annealing (b); Surface morphology of PFN-Br before annealing (c) and after 100 degrees annealing (d).


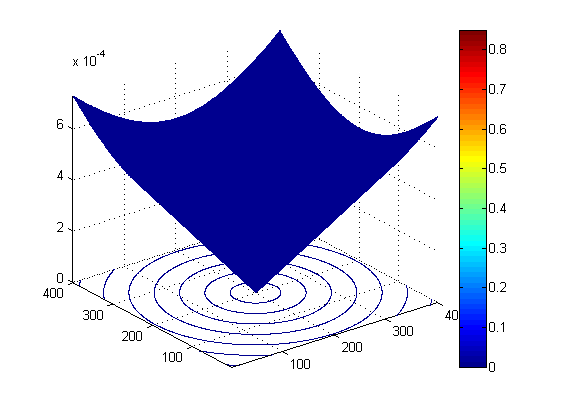


(a)


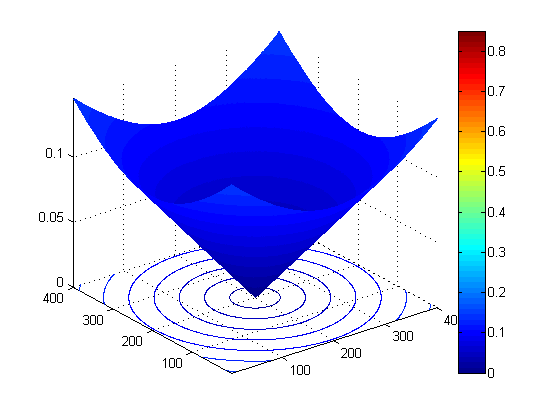


(b)


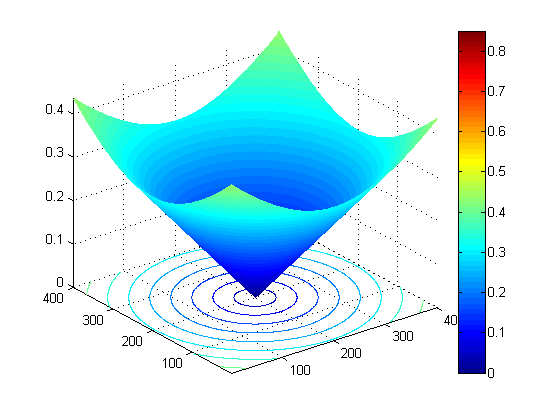


(c)


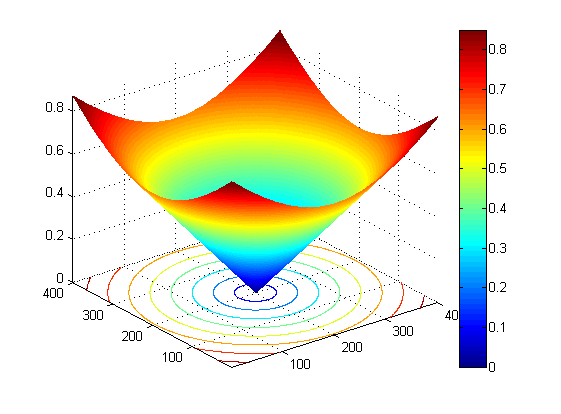


(d)

Figure S3 | Numerical simulation on dynamic magnetic fields at the exciton generation interface with frequency variation: (a) 50 Hz; (b) 10,000 Hz; (c) 30,000 Hz; (d) 60,000 Hz. The color bar shows the magnitude of self-generated magnetic fields. X and Y axis unit: 10^-5^ m; Z axis unit: mT.


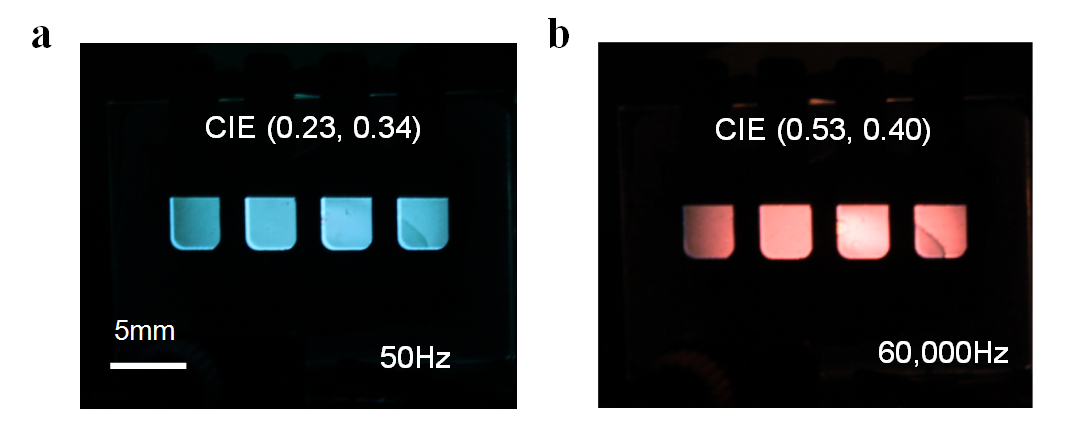


Figure S4 | Different color emissions of the pixels at 50 Hz (a) and 60 kHz (b).


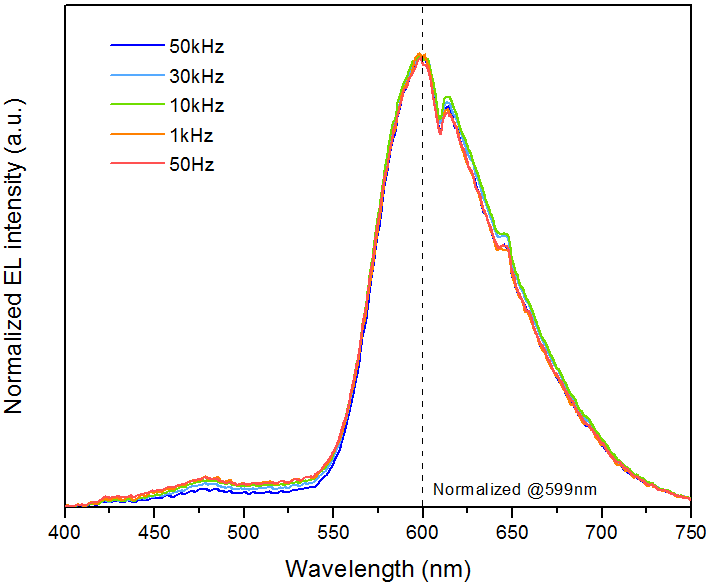


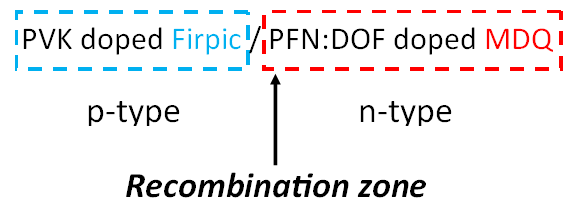


Figure S5 | EL spectrum of ITO/PEDOT:PSS doped ZnO NPs/PVK: 3 wt% Firpic/PFN:DOF: 3 wt% Ir(MDQ)_2_(acac)/TPBi/Al at driving frequency of 50 Hz, 1,000 Hz, 10,000 Hz, 30,000 Hz, and 50,000 Hz. No noticeable spectrum change or shift is found since the electron-hole recombination zone is fixed at the interface of PVK/PFN:DOF where a p-n junction is formed.


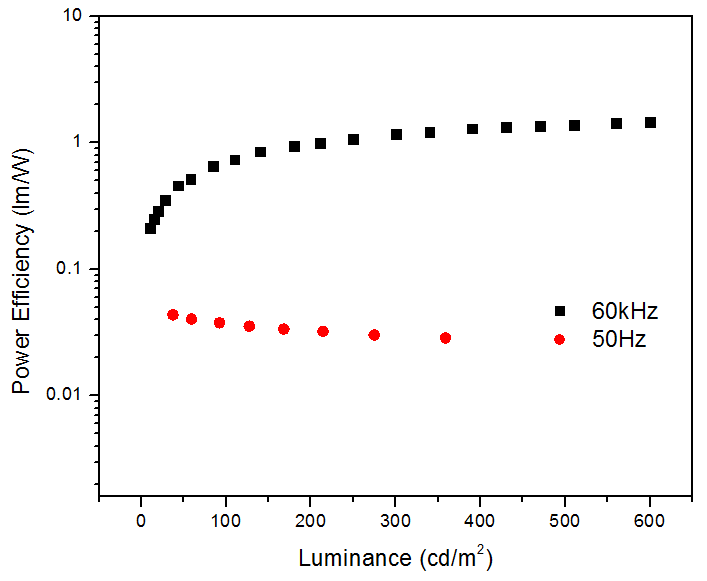


Figure S6 | The power efficiency of color tunable AC-OEL device at low and high frequency.


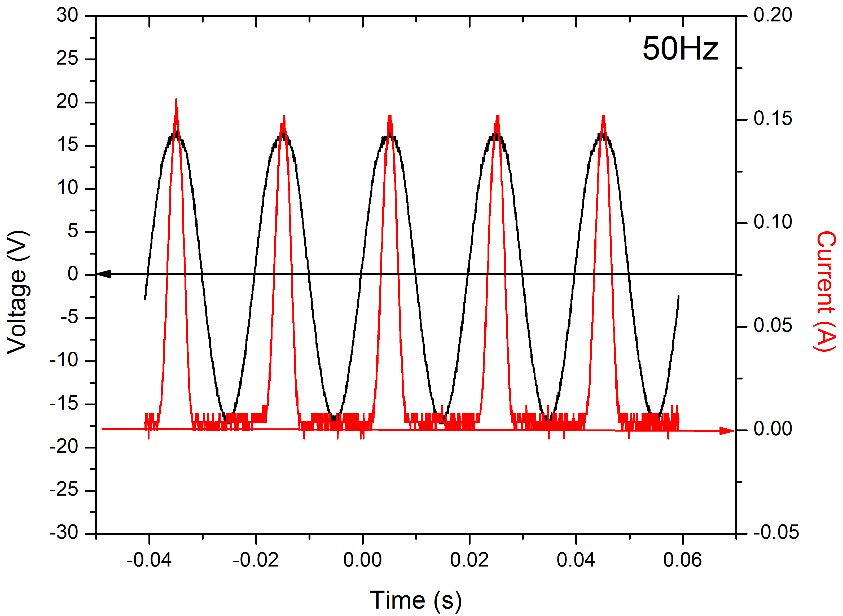


(a)


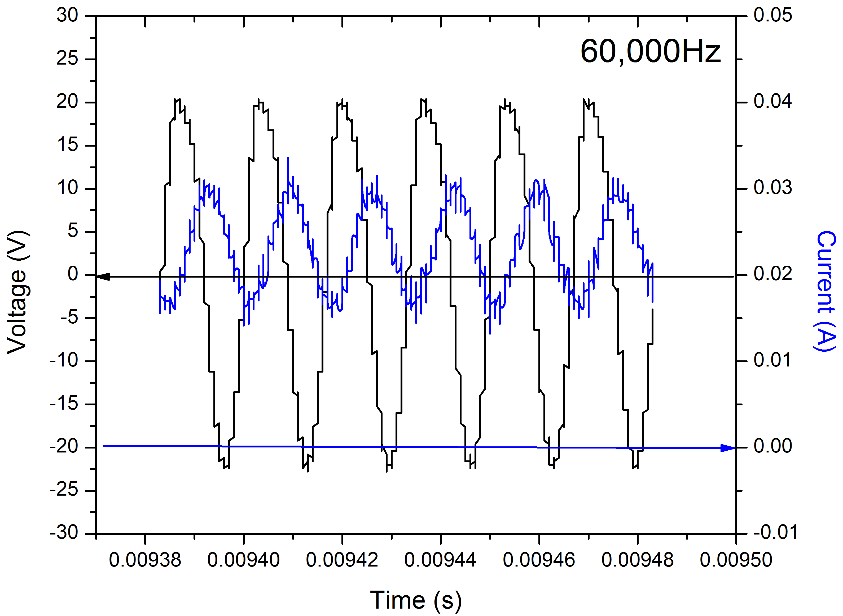


(b)

Figure S7 | Current and voltage transient properties of AC-OEL device at frequency of 50 Hz (a) and 60,000 Hz (b).


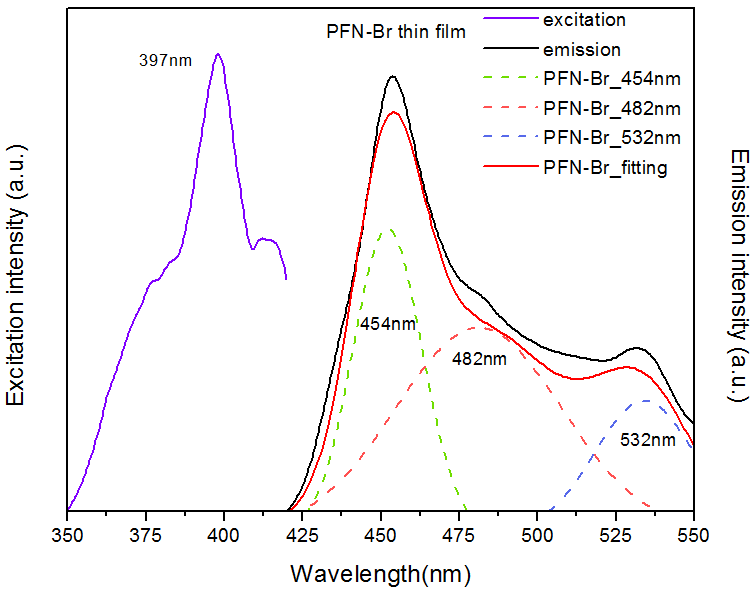


(a)


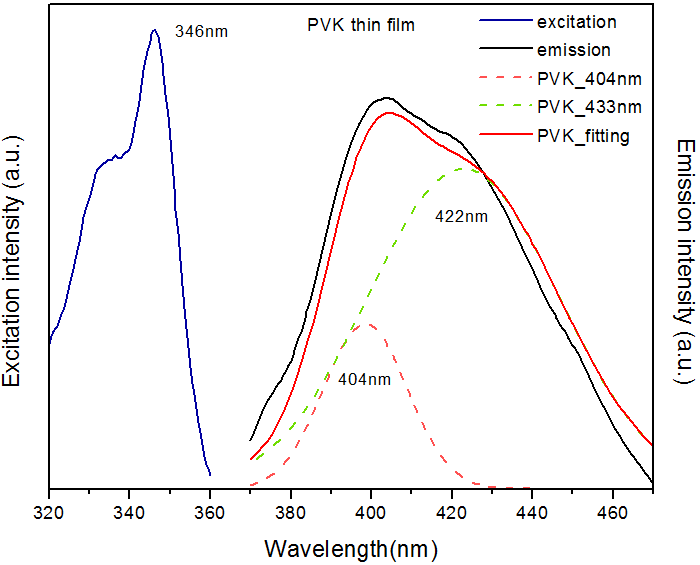


(b)


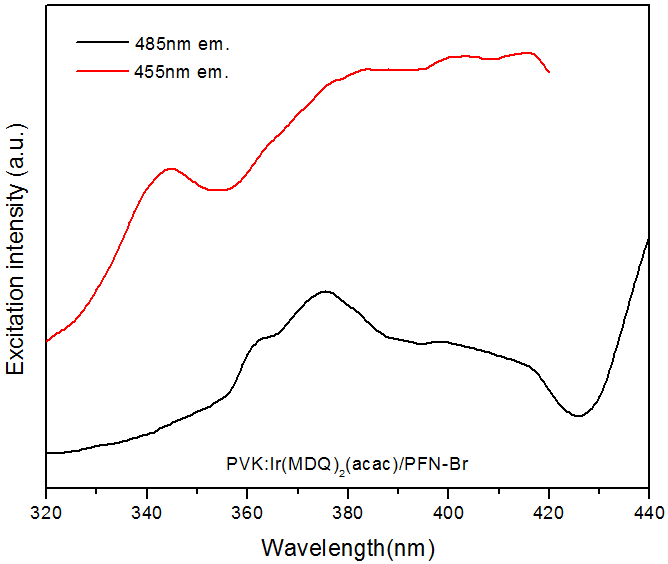


(c)


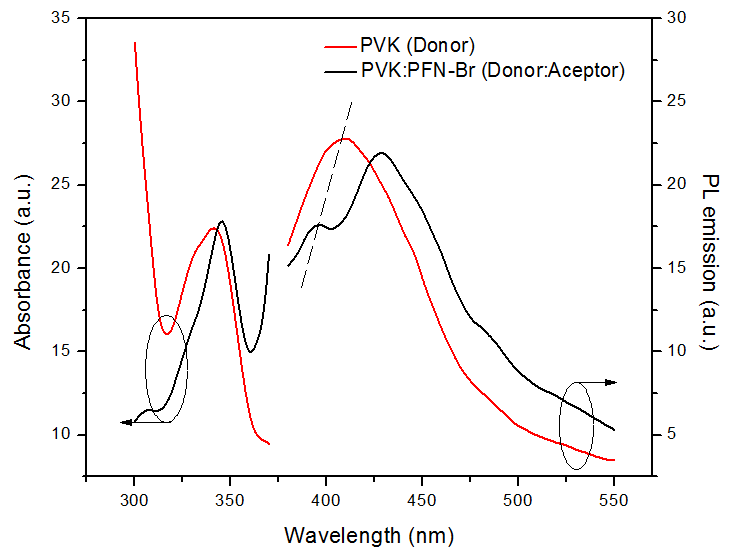


(d)

Figure S8 | Photoluminescence spectroscopy for organic functional materials. (a) Excitation and emission spectra for PVK film; (b) Excitation and emission spectra for PFN-Br film; (c) Excitation spectra for PVK: 3 wt% Ir(MDQ)_2_(acac)/PFN-Br complex emission unit. (d) Resonance energy transfer (FRET) efficiency measurement.


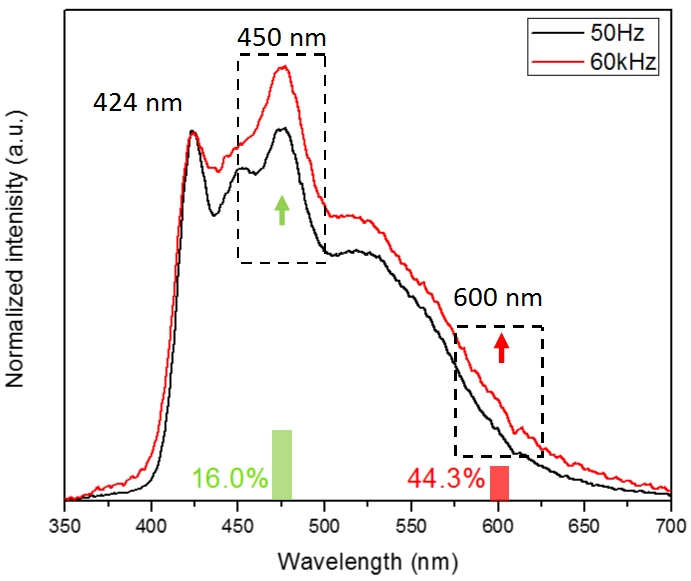


(a)


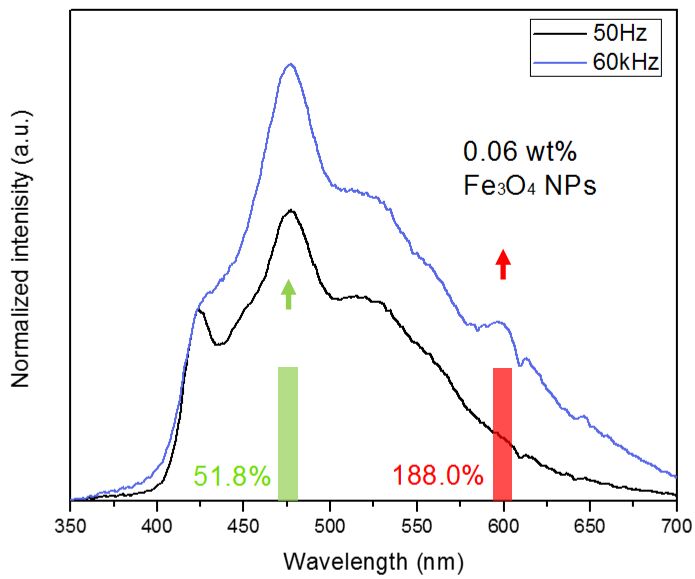


(b)


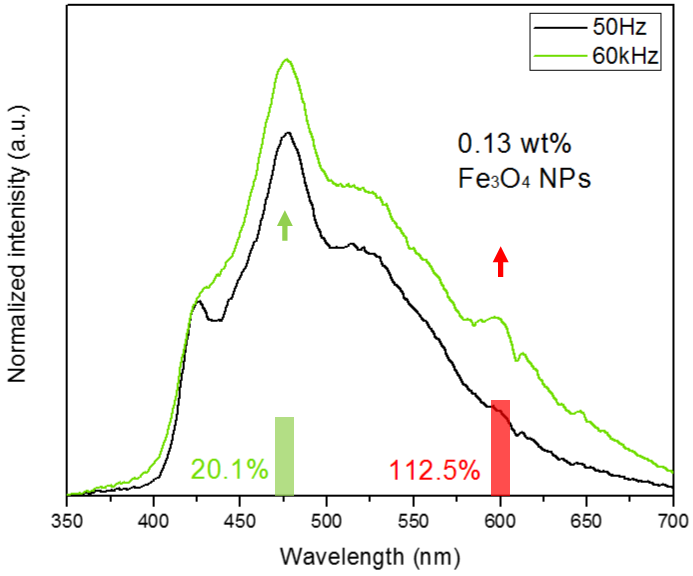


(c)


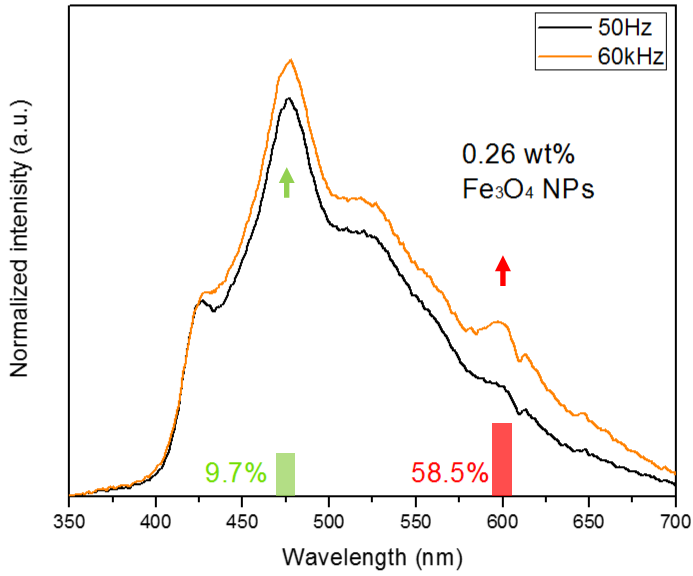


(d)

Figure S9 |Frequency sensitive EL spectrum of AC-OEL device with different doping ratios of Fe_3_O_4_ magnetic particles: (a) w/o, (b) 0.06 wt%, (c) 0.13 wt%, and (d) 0.26 wt%. The green bar and red bar in each graph represent the relative increase of 477 nm singlet emission and 600 nm phosphorescent peak, respectively. The percentages next are increase rates for fluorescent and phosphorescent emission in each case


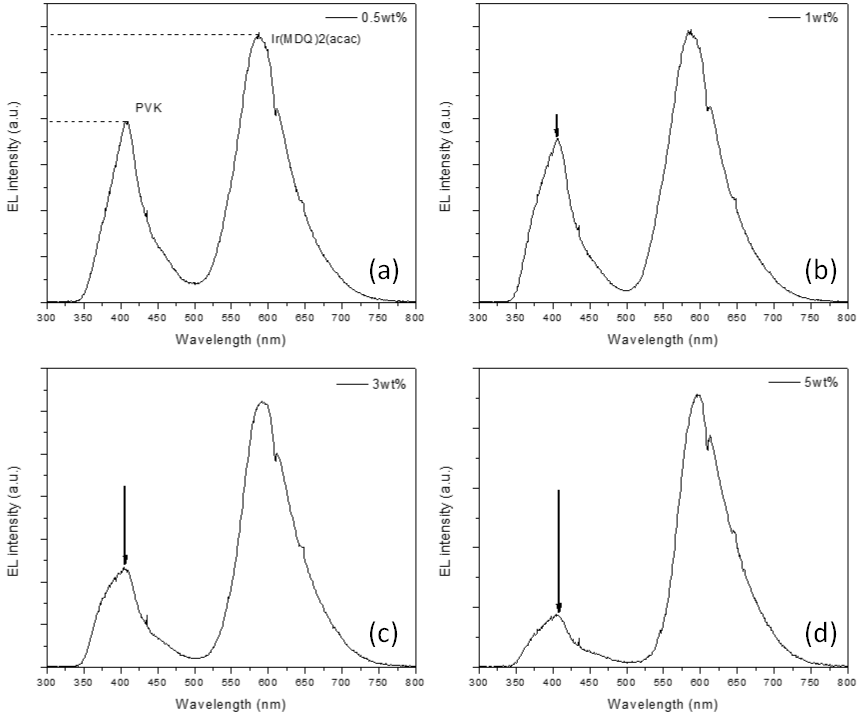


Figure S10 | EL spectrum of the AC-OEL devices with different concentrations of Ir(MDQ)_2_(acac) in PVK host: (a) 0.5 wt%, (a) 1 wt%, (a) 3 wt%, and (a) 5 wt%. Device structure: ITO/PEDOT: ZnO NPs /PVK: X wt% Ir(MDQ)_2_(acac) / TPBi/Al. The decreased PVK peak (~400 nm) with increasing dopant amount of irdium complex suggests that the Forster energy transfer between PVK to Ir(MDQ)_2_(acac) molecules are incomplete in the lower doping device.


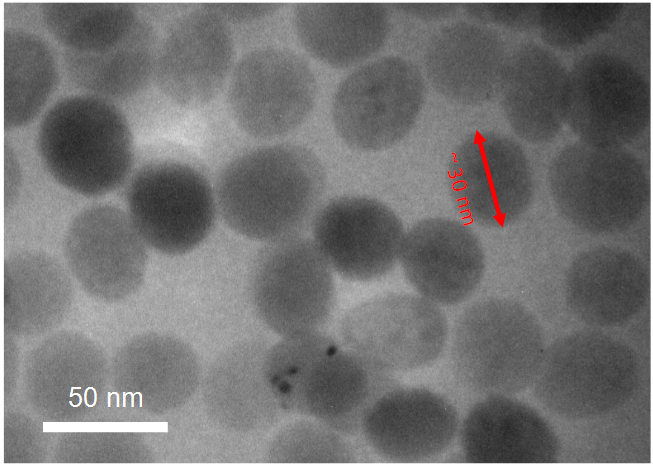


Figure S11 | TEM image of Fe_3_O_4_ magnetic nanoparticles (NPs) in PFN-Br polymer matrix.


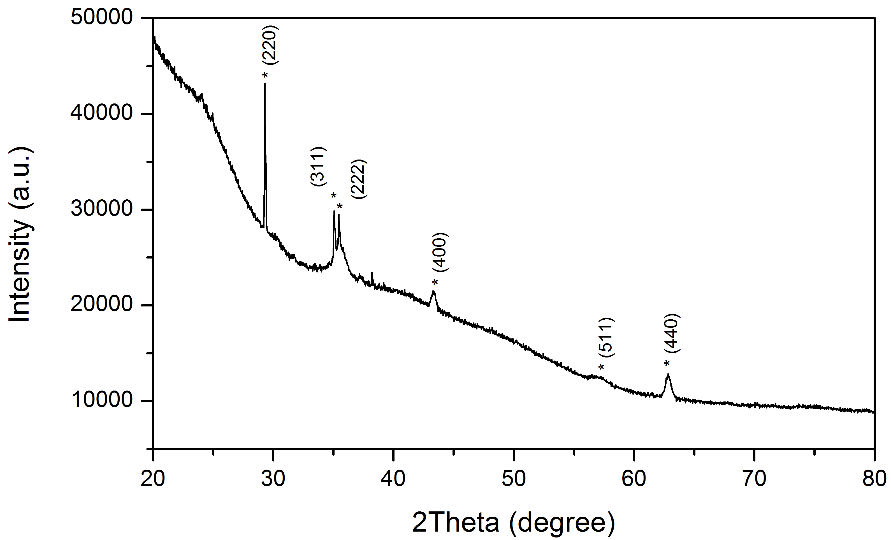


Figure S12 | XRD pattern of Fe_3_O_4_ magnetic nanoparticles (NPs).


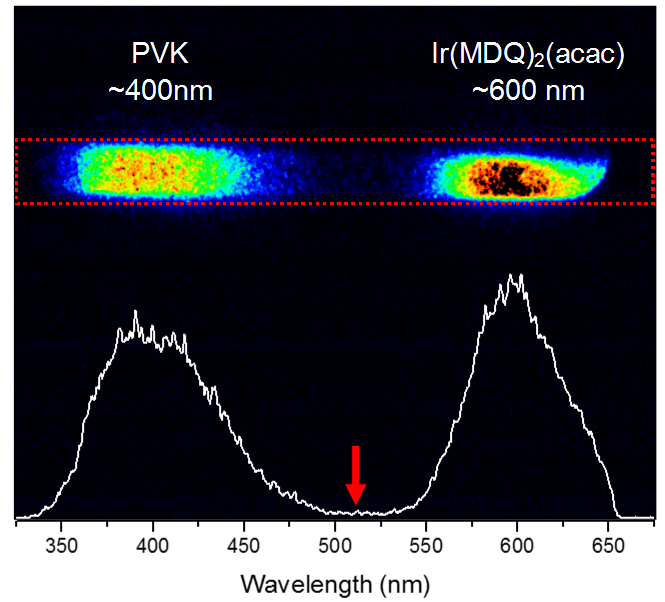


(a)


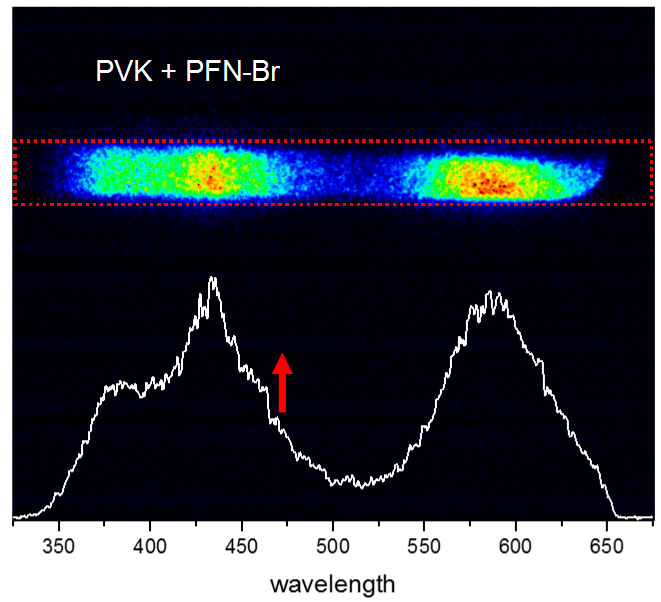


(b)


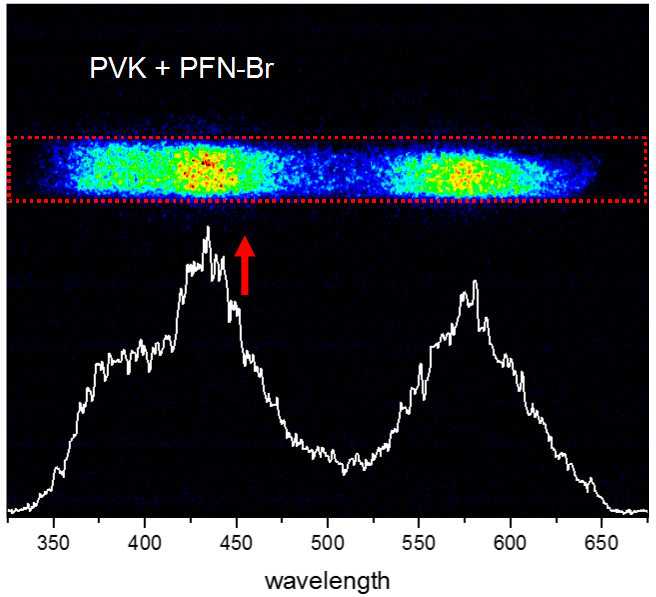


(c)

Figure S13 | Time integrated streak images of PVK:3 wt% Ir(MDQ)_2_(acac)/PFN-Br, PVK:3 wt% Ir(MDQ)_2_(acac)/PFN-Br:0.125 wt% Fe_3_O_4,_ PVK:3wt% Ir(MDQ)_2_(acac)/PFN-Br:0.375 wt% Fe_3_O_4_


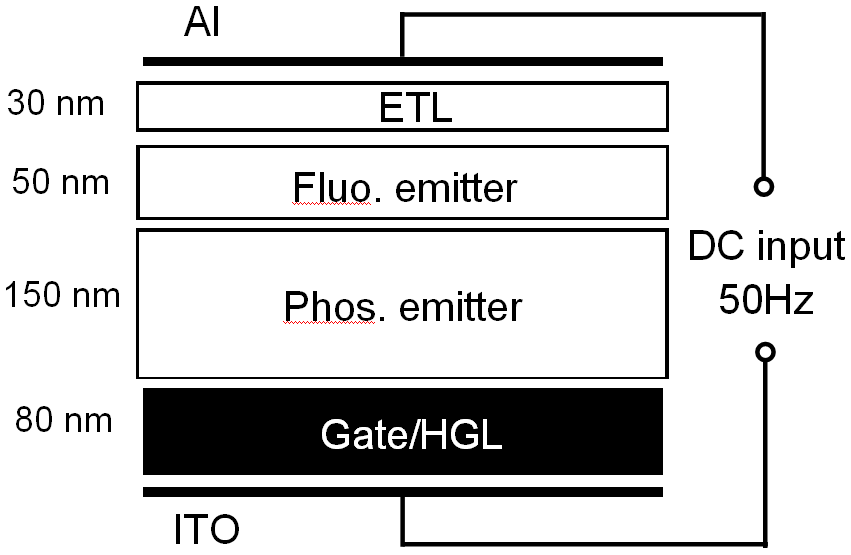


(a)


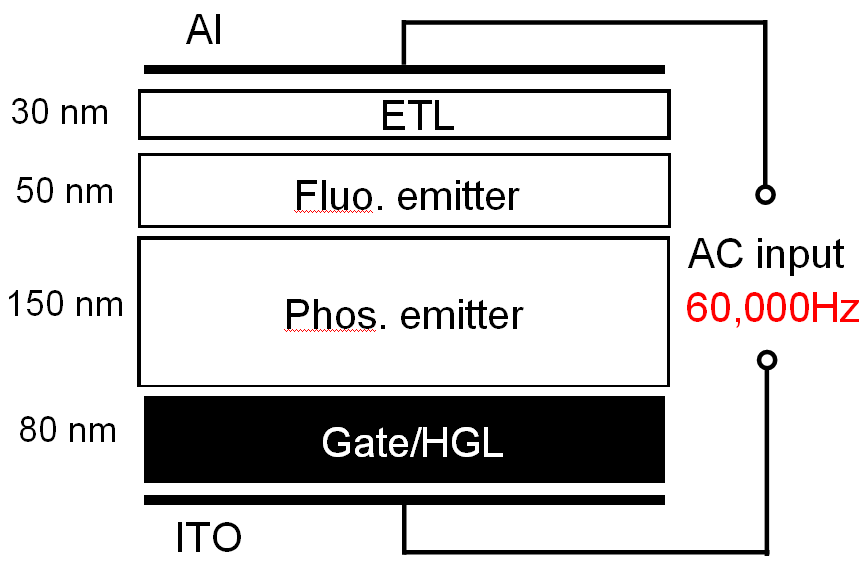


(b)


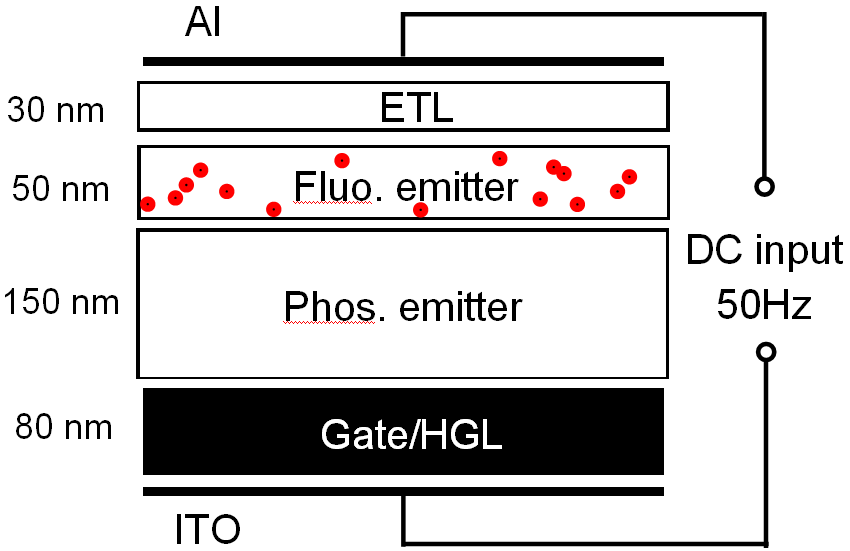


(c)

Figure S14 | Structural configurations of OEL device without magnetic field (a), with AC magnetic field (b), Fe_3_O_4_ magnetic nanoparticles magnetic field (c).


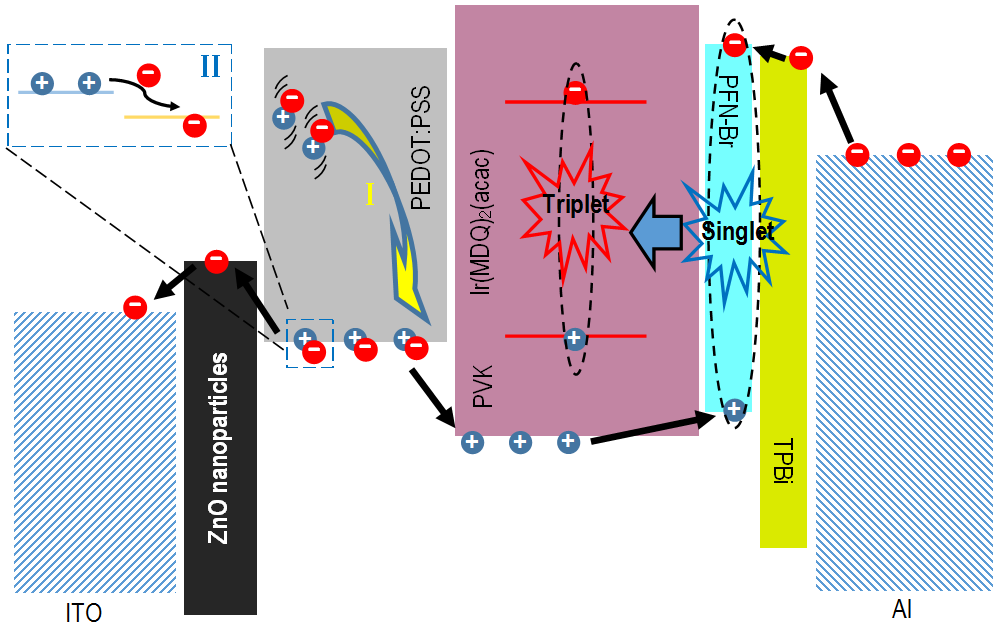


hole barrier:

0.2eV

electron barrier:

1.97eV

Figure S15 | Energy level diagram of AC-OEL device. The two free carrier sources are (I) Polarization current; (II) Electron-donor/electron-acceptor heterojunctions. Singlet-spin excitons are created in PFN-Br and transferred to PVK:Ir(MDQ)_2_(acac) because of excitons saturation.


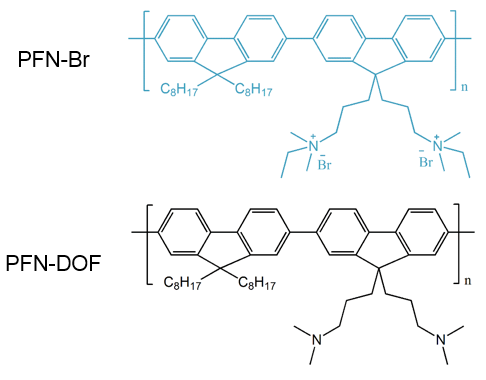


Figure S16 | Chemical structures of PFN-Br and PFN-DOF in comparison.


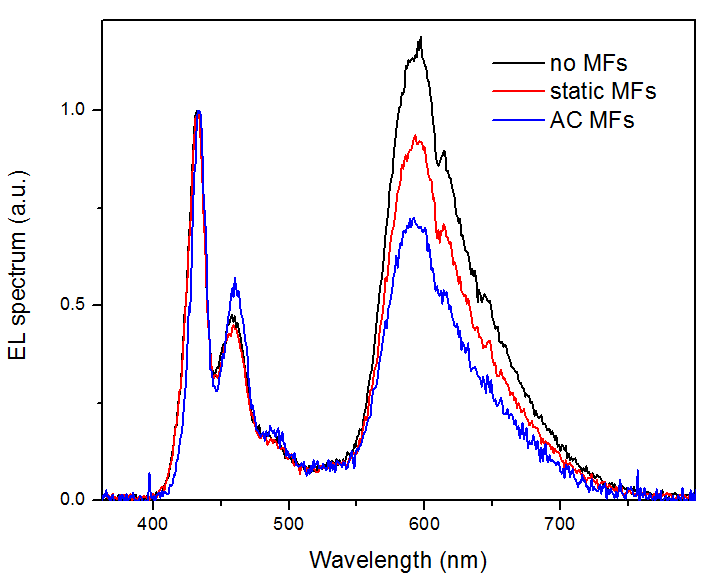


Figure S17 | EL spectrum of device without and with magnetic fields (static or AC) in a constant current density of 100 mA cm^-1^.


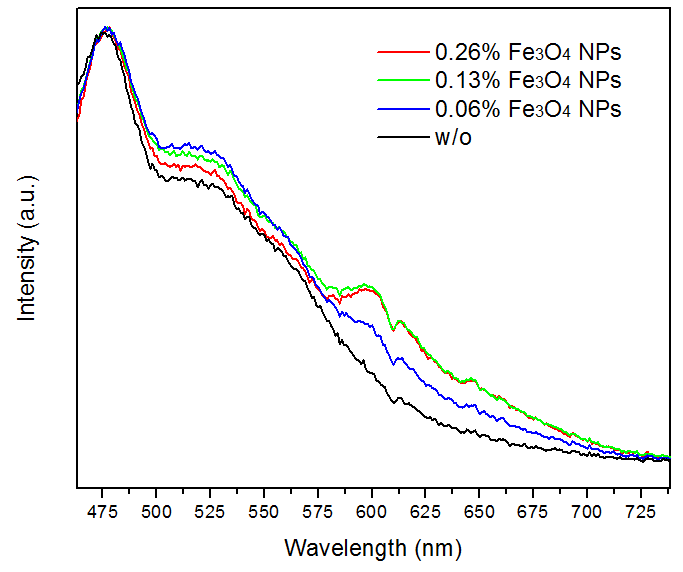


Figure S18 | EL spectrum of AC-OEL devices with a variety of Fe_3_O_4_ NPs concentrations.


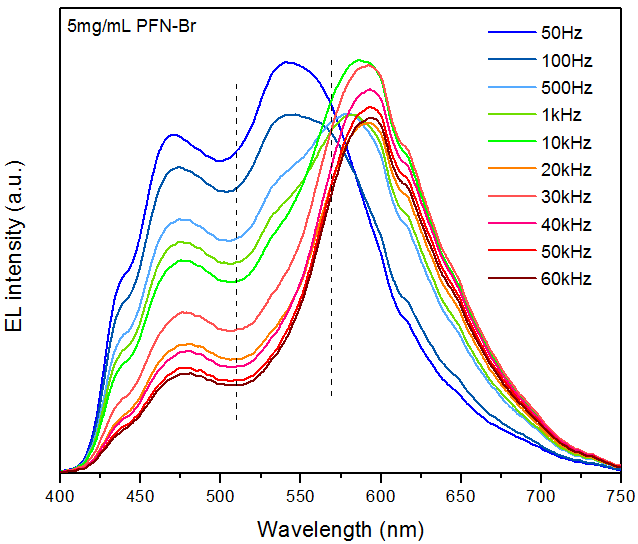


(a)


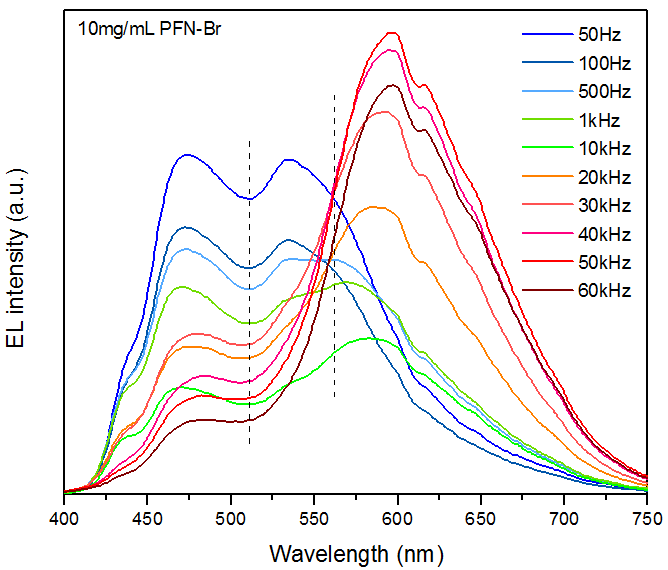


(b)

Figure S19 | EL spectra shift of AC-OEL devices with 30 nm (a) and 80 nm (b) PFN-Br spun-cast from Me(OH) solution.


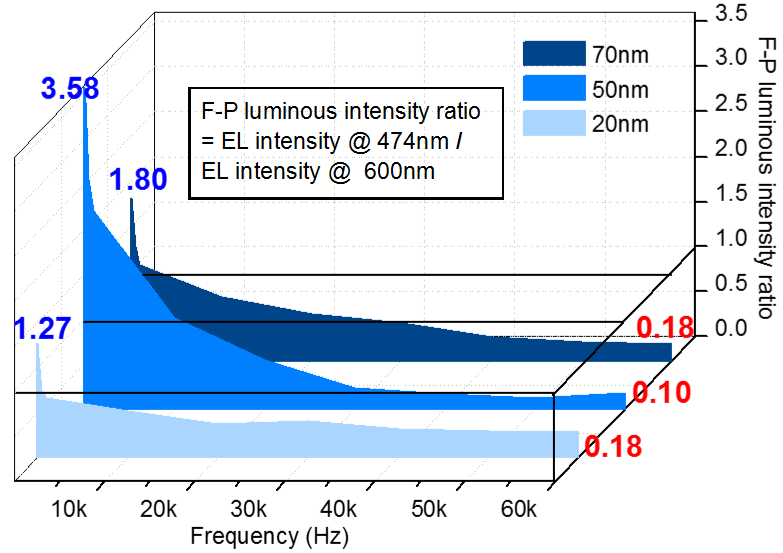


Figure S20 | Intensity ratio between 474 nm peak and 590 nm peak as a function of frequency in the PFN-Br’s concentrations of 20 nm, 50 nm, and 70 nm.


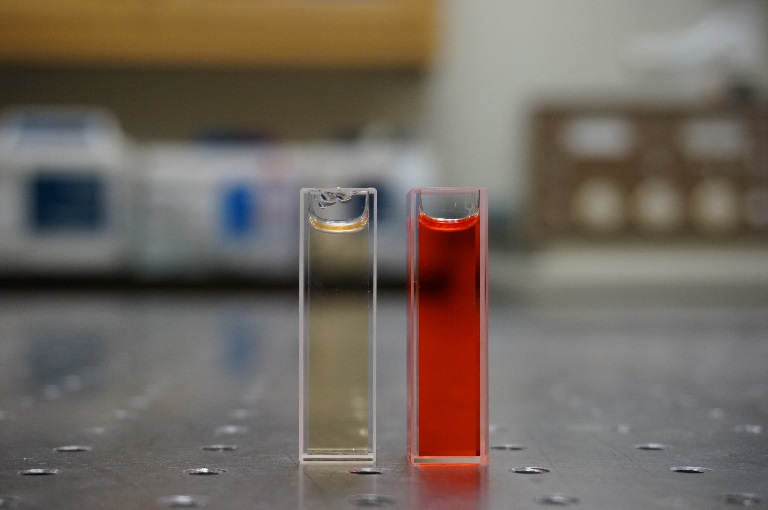


(a)


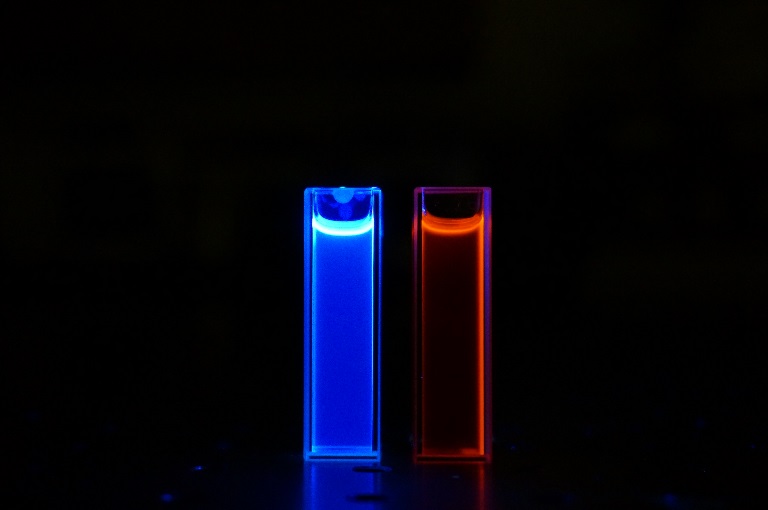


(b)

Figure S21 | Photoluminescence of Ir(MDQ)2(acac) and PFN-Br in solutions in room light (a) and with UV light (b).


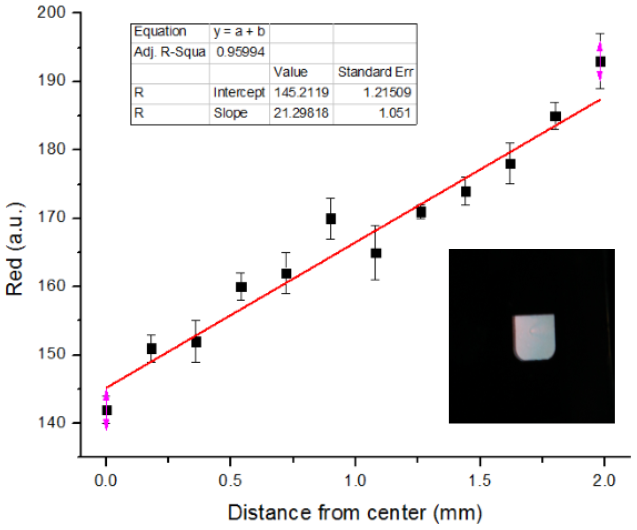


Figure S22 | Red color distribution over the distance from center. The central area (right side of the pixel) has the smallest red index (142) indicating a dominant blue fluorescent emission. The red index shows a linear increases to 193 away from the center, which is consistent with the expectation based on the non-homogenous film and the uniform AC magnetic field coupling mechanisms.

Table S1 | Materials used in this work.

| Abbreviation | Chemical name | Supplier |
| --- | --- | --- |
| ZnO NPs | Zinc oxide nanoparticles | Sigma-Aldrich |
| PEDOT:PSS | poly(3,4-ethylenedioxythiophene) polystyrene sulfonate | Heraeus |
| PVK | poly(N-vinylcarbazole | Sigma-Aldrich |
| Ir(MDQ)_2_(acac) | bis(2-methyldibenzo[f,h]quinoxaline) (acetylacetonate)iridium(III) | Lumtec |
| PFN-Br | poly[(9,9-bis(3'-((N,N -dimethyl)-N -ethylammonium)-propyl)-2,7-fluorene)-alt-2,7-(9,9-dioctylfluorene)] | Lumtec |
| PFN-DOF | poly[(9,9-bis(3'-(N,N-dimethylamino)propyl)-2,7-fluorene)-alt-2,7-(9,9-dioctylfluorene)] | Lumtec |
| Firpic | bis[2-(4,6-difluorophenyl)pyridinato-C2,N](picolinato)iridium(III) | Lumtec |
| TPBi | 2,2′,2"-(1,3,5-Benzinetriyl)-tris(1-phenyl-1-H-benzimidazole) | Lumtec |
| Fe_3_O_4_ NPs | Iron oxide nanoparticles | Sigma-Aldrich |

Movies S1 | The dynamic self-generated magnetic fields are coupled at the PVK/PFN-Br interface with strong electrical fields. The slow motion movie records the magnetic fields variation in 22.8 μs which is one and half period of 60,000 Hz. The proposed dimension of devices is 4 mm×4 mm. The amplitude of the magnetic field is 0.85 mT. As indicated, the direction of magnetic field is reversed alternatively between clockwise and counter clockwise. (Movie duration: 7 s)

Movies S2 | Demonstration of output spectrum shift from blue fluorescence to red phosphorescence with frequency. The driving frequency was continuously changed from 50 Hz to 60,000 Hz. The luminance was controlled at 250±50 cd/m^2^. (Movie duration: 33 s)
